# NGS of Virus-Derived Small RNAs as a Diagnostic Method Used to Determine Viromes of Hungarian Vineyards

**DOI:** 10.3389/fmicb.2018.00122

**Published:** 2018-02-06

**Authors:** Nikoletta Czotter, Janos Molnar, Emese Szabó, Emese Demian, Levente Kontra, Ivett Baksa, Gyorgy Szittya, Laszlo Kocsis, Tamas Deak, Gyorgy Bisztray, Gabor E. Tusnady, Jozsef Burgyan, Eva Varallyay

**Affiliations:** ^1^National Agricultural Research and Innovation Center, Agricultural Biotechnology Institute, Gödöllo, Hungary; ^2^Research Center of Natural Sciences, Institute of Enzymology, HAS, Budapest, Hungary; ^3^Department of Biotechnology, Nanophage-therapy Center, Enviroinvest Corporation, Pécs, Hungary; ^4^Department of Horticulture, Georgikon Faculty, University of Pannonia, Keszthely, Hungary; ^5^Department of Viticulture, Institute of Viticulture and Oenology, Szent-Istvan University, Budapest, Hungary

**Keywords:** grapevine, vineyard, virus, virome, diagnostics, metagenomics, small RNA, NGS

## Abstract

As virus diseases cannot be controlled by traditional plant protection methods, the risk of their spread have to be minimized on vegetatively propagated plants, such as grapevine. Metagenomic approaches used for virus diagnostics offer a unique opportunity to reveal the presence of all viral pathogens in the investigated plant, which is why their application can reduce the risk of using infected material for a new plantation. Here we used a special branch, deep sequencing of virus-derived small RNAs, of this high-throughput method for virus diagnostics, and determined viromes of vineyards in Hungary. With NGS of virus-derived small RNAs we could detect not only the viruses tested routinely, but also new ones, which had never been described in Hungary before. Virus presence did not correlate with the age of the plantation, moreover phylogenetic analysis of the identified virus isolates suggests that infections are mostly caused by the use of infected propagating material. Our results, validated by other molecular methods, raised further questions to be answered before this method can be introduced as a routine, reliable test for grapevine virus diagnostics.

## Introduction

Grapevine can host more than 60 viruses and viroids (Al Rwahnih et al., 2009; Martelli, 2014), often as multiplied infection. Vegetative propagation and long lifetime of the plantation increase the risk of virus infection which cannot be controlled by traditional plant protection methods. The use of highly adaptive cultivars globalizes not only the presence of the particular cultivar but also the spread of new pathogens and their vector organisms. Safety regulations deal only with a limited number of known viruses and ignore new invading pathogens, which can lead to the use of infected propagating material and produce a new level of persistent infection risk. Traditional diagnostic methods can only answer the question whether or not the investigated virus is present in our sample, and need preliminary information about the pathogen (antigen for ELISA and sequence information of the particular variant for PCR-based methods). In striking contrast, deep sequencing offers a unique opportunity to reveal any virus or viroid present in the sample, expected or not (Boonham et al., 2014). Indeed, different platforms were used for the description of new grapevine viruses (Martelli, 2014) and also to create the virome of a vineyard (Coetzee et al., 2010). During virus infection small interfering (si) RNAs having the same sequence as the infecting viruses are formed (Baulcombe, 2004; Molnar et al., 2005; Donaire et al., 2009; Kreuze et al., 2009; Szittya et al., 2010) by the RNA interference (RNAi) based defense reaction of the plant. Deep sequencing of the small RNA (sRNA) population extracted directly from field plants offers a unique opportunity in virus diagnosis to identify several variants of grapevine infecting viroids (Navarro et al., 2009) or viruses (Pantaleo et al., 2010) even if they are alien on the plant or have never been described before (Zhang et al., 2011; Giampetruzzi et al., 2012; Wu et al., 2012). In our work we used this cutting-edge technique, the deep sequencing of virus-derived siRNAs to reveal the sanitary status of vineyards in our country. Analysis of the sRNA sequence dataset obtained using our bioinformatics pipeline enabled us to describe viruses never before reported from our country (Grapevine Syrah virus 1 and Grapevine Pinot Gris virus, Grapevine Satellite virus). Beside these new descriptions we analyzed our samples for the most widespread viruses with RT-PCR (using published diagnostic primers) and sRNA NGS in parallel. In most cases our results could be validated, but we also found contradictions in several cases, which is also discussed.

## Materials and methods

### Plant material, sample preparation

Samples were collected from 14 vineyards representing 18 different varieties and nine wine-growing-regions of Hungary. Shoots of 1–10 randomly chosen individual plants per plantation were collected on the field or from sprouted canes. RNA was extracted from various organs: shoot tips, young leaves (until the 3rd internode from the shoot apex), older leaves (lower than the 3rd internode), tendrils, and inflorescenses by CTAB method (Gambino and Gribaudo, 2008). RNA pools representing each plant were generated mixing equal amounts of RNA originating from the different organs. These individual plant RNA pools (library 14–18) or a plantation pool generated by the same strategy, representing all of the sampled plants from the same plantation (library 1–13) was used for small RNA library preparation (18 libraries in total) and sequenced using single index on a HiScanSQ by UD Genomed (Debrecen, Hungary) 50 bp, single end (8 samples/1 sequencing lane). Fastq files of the sequenced libraries are deposited to the GEO and can be accessed through series accession number GSE106240.

### Pipeline for data evaluation of NGS results (bioinformatics)

The resulting reads were sorted according to their indexes. Adapters of the sequenced reads were removed by the Trimmomatic program (Bolger et al., 2014), their quality was checked by the FastQC program (http://www.bioinformatics.babraham.ac.uk/projects/fastqc) and deduplicated by the Picard MarkDuplicates tool (http://broadinstitute.github.io/picard). For virus detection we used two different pipelines in parallel: (A) Short reads were mapped to viral reference genomes (Refseq viral database of NCBI from only plant and invertebrate hosts were used) by the BWA-aln short read aligner (Li and Durbin, 2009) with default options. Mapped reads were counted both with and without deduplication using samtools idxstats (Li, 2011). Redundant reads of the resulted hits were equalized to read/million read. Consensus viral sequences from the aligned deduplicated reads were generated using the samtools/bcftools (Li and Durbin, 2009) pipeline. Coverage of the appropriate genome was counted as % of the genome covered by nucleotide information from the mapped small RNA reads. (B) De novo assembling of the deduplicated reads was performed using Velvet with k-mer: 13, 15, 17 (Zerbino and Birney, 2008). The generated contigs were annotated by BLAST megablast (Morgulis et al., 2008) to the RefSeq of NCBI.

### Sequence comparison

To compare consensus sequences of virus variants of the different libraries or sequenced PCR products we used the CLUSTAL Omega program (Sievers et al., 2011) and neighbor-joining algorithm implemented in MEGA v.6 (Tamura et al., 2013) Bootstrap values >70% (1000 bootstrap replicates) were used.

### Validation of predicted virus diagnostics by RT-PCR

cDNA was synthetized from pooled RNA extracts representing each plantation using random primer and the RevertAid First Strand cDNA Synthesis Kit (Thermo Fisher Scientific, USA) (according to the manufacturer's instructions). The cDNA generated was used as templates for PCR reactions using Phire Hot Start II DNA Polymerase (Thermo Fisher Scientific, USA) and published diagnostic primers or new ones (see Supplementary Table 5) designed according to the consensus sequence generated by mapping our small RNA reads to the reference genomes. To detect GRVFV, we used cDNA generated with a GRVFV-specific GRVFV-R/6391 primer. PCR products were analyzed by agarose gel electrophoresis. For Sanger sequencing cDNA was synthetized from pooled RNA extracts of individual plants and virus-specific PCR was done using Phusion Hot Start II High-Fidelity DNA Polymerase (Thermo Fisher Scientific, USA) or Q5 Hot Start High-Fidelity DNA Polymerase (New England Biolabs, UK) DNA polymerase. The purified products were cloned into pJET 1.2 vector (Thermo Fisher Scientific, USA) and sequenced. Sequences were deposited into GenBank (for GenBank Accession Numbers see Supplementary Figure 6).

### Validation by Northern blot

For Northern blot analyses 4–5 μg of total RNA was separated on formaldehyde-1.2% agarose gel and blotted to Amersham Hybond-NX membrane (GE Healthcare, UK), by capillary method using 20xSSC. Hybridization was carried out at 65°C in Church buffer (0.5 M Phosphate buffer, pH 7.2 containing 1% BSA, 1 mM EDTA, 7% SDS) overnight with the appropriate radioactively labeled probe, washed for 5 min in 2 × SSC, 0.1% SDS and for 15 min in 0.5 × SSC, 0.1% SDS at the temperature of the hybridization and exposed to an X-ray film.

Virus-specific, P^32^-labeled, DNA probes were prepared by using the DecaLabel DNA Labelling Kit (Thermo Fischer Scientific, USA). As a template we used the PCR-amplified and purified product of cloned region of viral genome. The virus piece cloned was a 1663-bp part amplified with GPGV5557F and GPGV7220R for GPGV, a 1324-bp product amplified by RBDV_RNA1F_4082 and RBDV_RNA1R_5406 for RBDV RNA1, and a 927-bp product amplified by GSVsatF72 GSVsatR999 for GSV.

## Results and discussion

### Sample collection and sequencing

As a survey to detect virus infections in Hungarian vineyards samples were collected directly from the field, in a random fashion, independently of any apparent symptom, in May 2014 or from sprouts of single bud cuttings. 14 vineyards differing in the variety grown and the age of the plantation from 9 wine-growing regions of the country were sampled (Supplementary Figure 1 and Supplementary Table 1, 2). Small RNA libraries were prepared from pooled samples representing either the plantation (libraries 1–13), or different varieties at the same plantation (library 14–18) and sequenced.

### Initial statistics

As a result of sequencing 8–14 million raw reads/library were generated (Supplementary Table 3). After trimming of the adapters, duplicates were removed and non-redundant reads (560000-1.6 million/library), without removing grapevine-specific sRNAs, were used for virus diagnostics. In different libraries 3.3–13% of the total non-redundant reads and 2.3–11.7% of the total redundant reads were mapped to viral reference genomes, representing 2–13 different viruses and viroids.

### Size distribution of sequenced sRNAs

**Figure 1:**
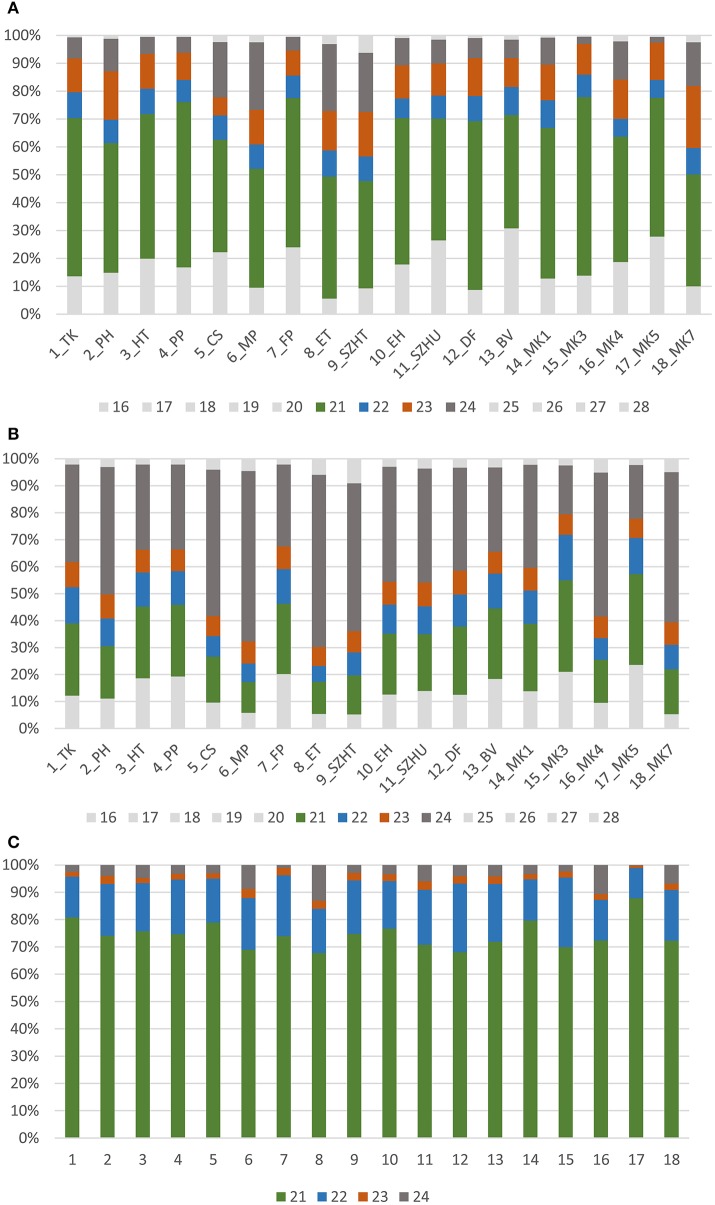
Size distribution of **(A)** trimmed, **(B)** non-redundant, **(C)** viral, redundant sequenced reads of the sequenced libraries.

Size distribution of redundant sRNA sequences showed that the majority of the reads was between 21 and 24 nt, indicating that the library preparation was successful (Figure 1A). Most of the reads were 21 nt long and contained miRNA sequences in accordance with our previous report (Pantaleo et al., 2010). As for non-redundant reads the 24 nt-long size class was overrepresented (Figure 1B), likely responsible for transcriptional gene silencing (TGS) (Borges and Martienssen, 2015). sRNAs are products of different plant DICERs (DCL1,−2,−3, and 4) and each DCL enzyme activity produces a specific size class. The products of DCL1 and DCL4 are 21 nt-long, whereas DCL2 generates 22 nt-long, and DCL3, 24 nt-long sRNAs. Whereas DCL1 has a key role in miRNA biogenesis, DCL3 generates siRNAs for TGS against parasitic nucleic acids (e.g., transposons) (Parent et al., 2012). The *Vitis vinifera* genome encodes four DCLs homologous to DCLs characterized in *Arabidopsis thaliana* (Zhao et al., 2015). Although according to that work VvDCL1 contains only one RNaseIII domain, VvDCL2 and VvDCL3 lacks a dsRB domain, moreover VvDCL4 lacks a PAZ domain, we think they must be fully functional since all characteristic sRNA size classes (21, 22, and 24 nt) were present in our samples. Figures 1A,B show the size distribution of all host-derived sRNA reads.

During antiviral silencing DCL4 and DCL2 process virus-derived dsRNAs into sRNAs. Virus- and viroid-specific sRNAs in our samples were almost exclusively 21–22 nt long, supporting the idea that in grapevine DCL2 and DCL4 are the key enzymes in virus-derived sRNA biogenesis (Figure 1C).

### Origin of viral SRNAS

In order to identify viruses present in our plantations, sRNA reads were aligned and mapped to reference genomes of all known viruses of plant or insect host origin. Coverage (in %) of the whole viral reference genome was also calculated. During this analysis virus-specific contigs were assembled with different k-mers (kmer13, 15, 17), and the resulting contigs were also aligned to this set of reference genomes. In Supplementary Table 4 the results of the bioinformatics analysis for quarantine viruses: Grapevine fanleaf virus (GFLV), Arabis mosaic virus (ArMV), Grapevine leafroll-associated virus 1-3 (GLRaV1-3), Grapevine virus A (GVA), Grapevine virus B (GVB), Grapevine fleck virus (GFkV), together with Grapevine chrome mosaic virus (GCMV), Grapevine red globe virus (GRGV), Grapevine asteroid mosaic-associated virus (GAMaV), Grapevine vein feathering virus (GRVFV), Grapevine Syrah virus 1 (GSyV1), Grapevine rupestris stem pitting-associated virus (GRSPaV), Grapevine Pinot gris virus (GPGV), Raspberry bushy dwarf virus (RBDV), Grapevine satellite virus (GSV) and viroids: Hop stunt viroid (HSVd) and Grapevine yellow speckled viroid 1-2 (GYSVd-1 and 2) are summarized. A virus or viroid was diagnosed as present if any virus/viroid specific contigs (if any with any kmer) was present, and coverage of the viral genome by small RNA reads was higher than 40% (in case of viruses) or 80% (in case of viroids). According to these results the tested plantations are free from GFLV, ArMV, and GLRaV2, but we usually found simultaneous presence of up to 13 of different viruses and viroids in the same plantation.

### Validation of deep sequencing results

**Figure 2:**
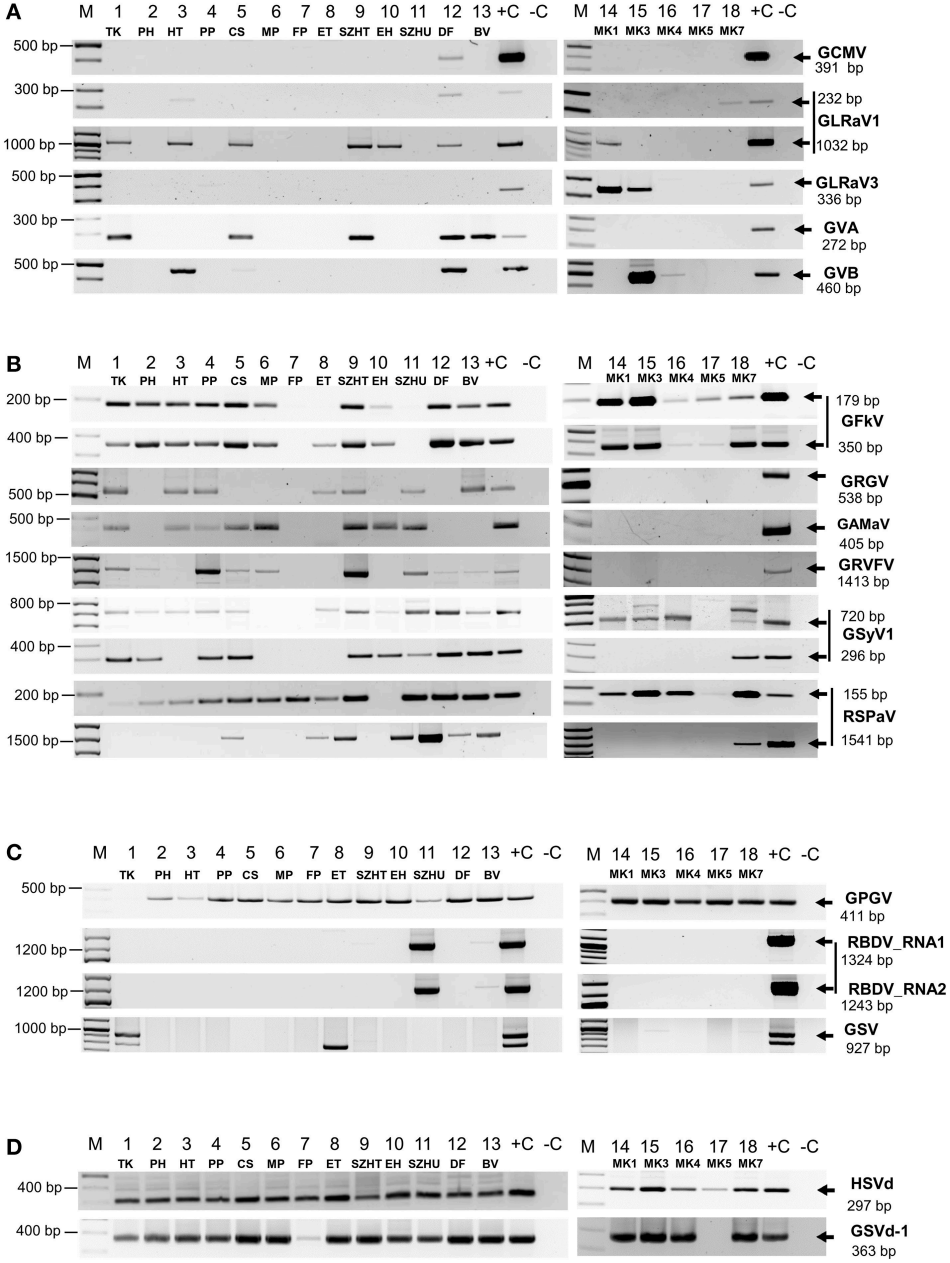
RT-PCR validation of sRNA NGS for **(A)** *Nepo*-, *Leafroll*-, and Vitiviruses, **(B)** Tymoviruses, **(C)** viruses which presence is not routinely tested and **(D)** viroids. cDNA was synthetized from pooled RNA extracts representing each vineyard using random primer and used as templates for PCR reactions with published diagnostic primers or new ones designed according to the consensus sequence generated by mapping our small RNA reads to the reference genomes. To detect GRVFV we used cDNA generated with a GRVFV-specific primer. PCR products were analyzed by agarose gel electrophoresis. (M), GenRuler 100 bp+; (+C), cDNA containing the tested virus was used as positive, or (–C), water as negative control.

In order to validate our deep sequencing results we synthetized cDNA from RNAs representing plantation pools and set up PCR reactions by published diagnostic primers or with primers designed according to the sequenced sRNA reads (Supplementary Table 5). Positive controls (cDNA from virus-containing samples) and negative controls were always included. PCR products were analyzed by separation on 1.2 % agarose gels (Figure 2) and traditional Sanger sequencing. Results of the sRNA NGS virus diagnostics and its comparison with RT-PCR are summarized in Table 1. Sequences were deposited to GenBank and used for phylogenetic comparison (see Supplementary Table 6 for summary). Our results showed that the reliability of sRNA NGS as a diagnostic tool varied from virus to virus, and we discuss it for each of the identified viruses.

**Table 1:**
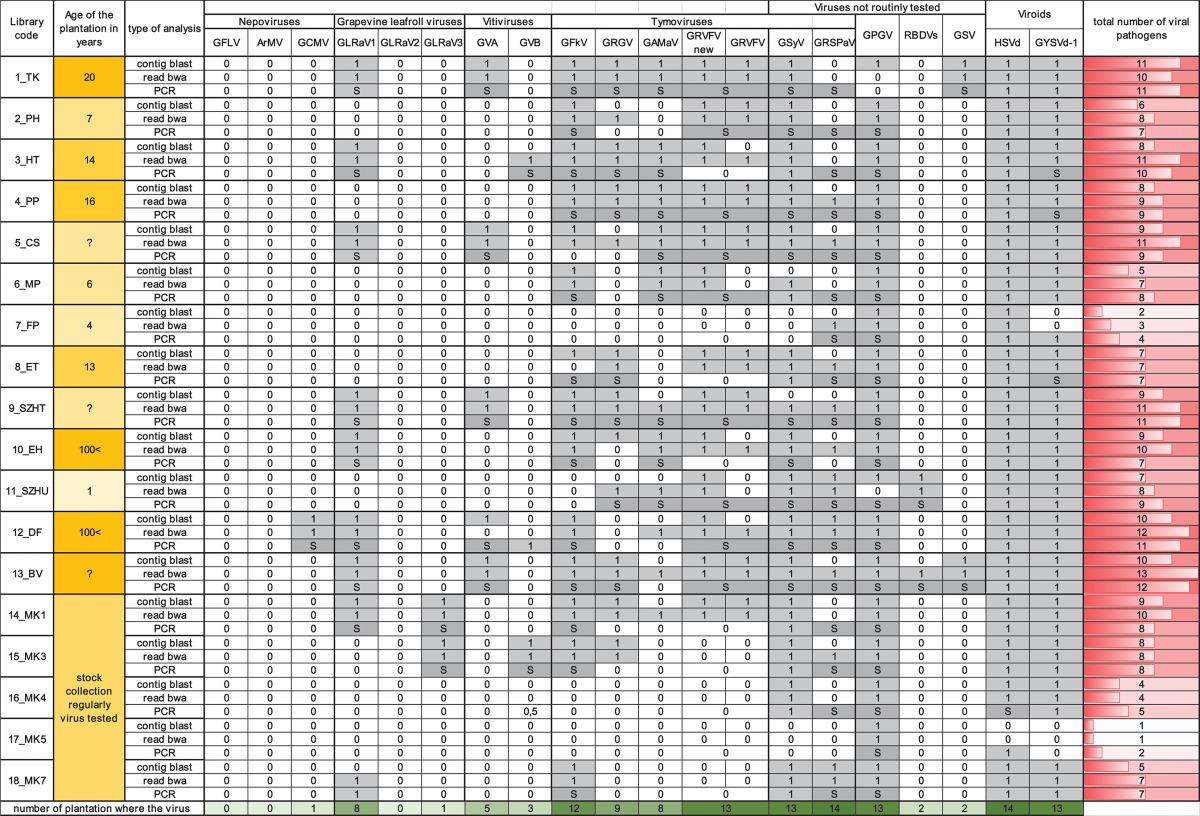
Summary of the small RNA NGS virus diagnostics and its RT-PCR validation for each library.

*Result of the bioinformatics analysis (contig blast and read bwa) was marked by 0 or 1. 0 was used if there was no hit for the appropriate virus, whereas 1 was used if any contigs could be built, or the coverage of the virus genome was higher than 40% (in case of viruses) or 80% (in case of viroids). Result of RT-PCR validation was marked as 1, if PCR product was present or by S if not only the product was present but it was Sanger sequenced. Any hit present was highlighted with gray color. Summary of the total number of viral pathogens in the library, and number of the plantations where the virus is present, was highlighted with red and green color respectively*.

### Nepoviruses

The only *Nepovirus* detected was GCMV present in 12_DF where partial CP could be amplified and sequenced (Figure 2A) (accession number MF100927). Interestingly enough, all of the GCMV RNA2 sequences in GenBank originated from Hungarian accessions (Elbeaino et al., 2014), and are more closely related to each other (more than 96% identity in this CP region) than to the isolate what we found (88–89% identity compared to other GCMV RNA2 sequences in this CP region) (Supplementary Figure 2A). The geographical origins of these Hungarian GCMV accessions are unknown. According to a recombination analysis of full GCMV RNA2 sequences, GCMV suggested to be a putative interspecies recombinant of GARSV and TBRV (Digiaro et al., 2015), which question could be further investigated incorporating HUDF isolate, however for its recombination analysis full RNA2 must be sequenced.

### Grapevine leafroll associated viruses

GLRaV1 was detected in nine of our samples, but validation with published diagnostic primers failed in six samples (Figure 2A, Table 1). Testing the assumption that diagnostic primers were designed to variable regions, new primers were designed to the HSP70 coding region based on the sequenced sRNAs reads. With this new set of primers we could successfully validate the presence of GLRaV1 in five additional libraries (Figure 2A). In 13_BV we could only detect the virus in two of the individuals (Supplementary Figure 3A), which might explain the lack of the PCR product in the pool. Phylogenetic analysis of our isolates showed that they clustered into two distinct groups, the same E and A as was suggested by Kominek et al. (2005) (Supplementary Figure 2B) and are only 82–92% identical to the reference genome, supporting the operation of high-level variability, due to which GLRaV1 can be easily overlooked by traditional diagnostic methods (Esteves et al., 2013). The only Hungarian GLRaV1 isolate (CSE_6.4.1.H) in GenBank (Cseh et al., 2013) (clustered to group E) was collected at the same region of the country from where HUTK and HUHT (clustered to group A) originated, suggesting that the source of the infection is more likely the propagation material. These results show that detection of GLRaV1 by sRNA NGS seems reliable, but its validation by RT-PCR may be problematic due to the high variability of the virus.

For GLRaV3 the result of sRNA NGS and its validation by RT-PCR using published diagnostic primer pairs correlated well (Table 1), detecting its presence in 14_MK1 and 15_MK3. Phylogenetic analysis of the sequenced part of the CP showed that these isolates (HUMK1 and HUMK3) are very closely related (98% identity on nt level) but share only 91% identity with the NY1 Reference genome from the USA (Supplementary Figure 2C). They clustered together with isolates from Brazil, Israel, and South Africa, but share only 90–91% identity with European strains. Their phylogenetical relationship based on the HSP70 region showed that they cluster into two distinct group together with isolates from different regions of the country (Cseh et al., 2013) (Supplementary Figure 2D). Samples 14_MK1 and 15_MK3 are different varieties in different rows of the same plantation. As these geographically linked variants tended to be divergent, it seems possible that the infection originated from an infected propagation material and is not the result of an onsite infection.

### Vitiviruses

GVA was detected at five, whereas GVB at two plantations (Table 1,Figure 2A). In 12_DF we could clone an RT-PCR product from both of these viruses although we failed to detect GVB by sRNA NGS. Testing individuals for their presence we have found that in this sample only one plant was infected (Supplementary Figures 3B,C), which could decrease the concentration of the virus below the detection limit by sRNA NGS, in the pool. GVA isolates were only 85–90% identical to the Italian Reference genome and grouped into two distinct clades within Group I, together with other European strains (Goszczynski, 2014) (Supplementary Figure 2E). GVB isolates showed higher variation: they were only 85% identical to each other and grouped into different clades (Fonseca et al., 2016) (Supplementary Figure 2F). We can conclude that sRNA NGS based virus diagnostics worked well for vitiviruses, but our results showed that using plantation pools containing extracts of non-infected plants can lower the virus concentration, and without further investigation slight and uneven infections can easily be overlooked.

### Tymovirales

#### Grapevine fleck virus (GFKV)

GFkV was one of the most widespread viruses present in 14 samples. Virus diagnostics by sRNA NGS and RT-PCR validation for GFKV presence usually correlated well (Table 1,Figure 2B). Although using diagnostic primers we failed to validate its presence in 8_ET, validation was successful with a new set of primers, designed according to the sequenced sRNA reads (Figure 2B). Sequence comparison of the isolates showed high variability: they were 85–95% identical to the Italian reference (Supplementary Figure 2G). Sequencing of the cloned PCR product in 5_CS showed that this is a product of GRVFV (MF461275) and not of GFKV, highlighting the high-level identity of these two viruses. However, validation of the presence of GFKV failed, it is possible that sequencing more clones would have yielded a GFKV-specific product.

Tymoviruses, involved in fleck complex, are closely related; they can coexist in the same plant and GFKV is often presents in co-infection together with GRGV, GAMaV, GRVFV (Sabanadzovic et al., 2000; Cretazzo et al., 2017) and sometimes with GSyV1, therefore it is not surprising that we could also detect these viruses in most of our samples.

#### Grapevine red globe virus (GRGV)

GRGV was detected in many of our samples but its presence could only be validated in seven libraries (Table 1,Figure 2B). GRGV has been identified in different parts of Europe (Sabanadzovic et al., 2000; Beuve et al., 2015; Cretazzo et al., 2017; Voncina et al., 2017) and also in California (El Beaino et al., 2001) and in Brazil (Fajardo et al., 2017). We detected GRGV in different regions of the country; the sequenced strains have 87–95% identity with the Reference strain (NC_030693) and clustered separately (Supplementary Figure 2H). Variability of different isolates and the fact that GRGV was not found in Czech and Slovak accessions—not even with NGS (Eichmeier et al., 2016)—further supports the idea that it is not originally present in Central-Europe, and possibly originates from infected propagation material of a geographically different origin.

#### Grapevine asteroid mosaic associated virus (GaMaV)

We could detect GAMaV in eight samples (Table 1,Figure 2B). Although described and well-known since 1994 (Boscia et al., 1994), there is still only limited sequence information about GAMaV. The Reference genome was only uploaded to GenBank in 2016 (NC_031692), but since that time—thanks to NGS surveys - it has been reported from Canada (Xiao and Meng, 2016) and from France (Candresse et al., 2017a). Our isolates are 94–96% identical to the Reference and 93–96% identical to each other, showing less variability than GRGV. As Hungarian GAMaV isolates clustered with isolates from different geographical origins (Supplementary Figure 2I), the use of virus-infected propagation material is the most straightforward explanation for their presence.

#### Grapevine rupestris vein feathering (GRVFV)

GRVFV was detected in 11 and 13 libraries using a full genome (AY706994) and a Reference Genome (NC_034205) (Reynard et al., 2017) for its detection respectively (Table 1 and Supplementary Table 4). The new Reference is only 77% identical to the first, Californian full genome, which shows the very high variability of this virus and the reason why the validation of its widespread presence has usually been failed (Pantaleo et al., 2010; Reynard et al., 2017). Our RT-PCR validation using cDNA produced by random probe also failed in all cases. We tried to increase the concentration of virus specific cDNA using virus-specific primer for cDNA synthesis and could amplify GRVFV in 9 libraries, but still failed in four samples (Figure 2B). Our isolates share 79–96 and 79–87% identity with AY706994 and NC034205, respectively, further confirming the diversity of this virus. Moreover, sequencing different individuals from the same plantation (HUCS, HUPP, HUTK, HUDF) revealed the presence of distinct variants (78–92% identical to each other) at the same plantation, clustering separately (Supplementary Figure 2J). This case shows that sRNA NGS has difficulties in accurate diagnostics of viruses with high variable genomes, however the presence of a Tymovirus could be accurately detected.

#### Grapevine Syrah virus 1 (GSyV1)

As we have reported previously we have found GSyV1 in Hungarian vineyards (Czotter et al., 2015 present in 15 libraries (Table 1). With DetF-DetR (Al Rwahnih et al., 2009#3) amplifying putative MP, we could validate its presence in 10 while with primers amplifying part of the CP (Sabanadzovic et al., 2009) in further six samples (Figure 2B). High prevalence of GSyV1 have also been found in Czech and Slovak grapevines which also showed high variability at the 5' putative MP coding region (Glasa et al., 2015), resulting false negative result if the above primers were used for diagnostics. Our isolates grouped distantly both into the two major and the diverged third clades suggested by Glasa (Glasa et al., 2015) (Supplementary Figure 2K). Moreover sequences of GSyV1 from different individuals of the same plantation (HU11TK2 and HU11TK9) showed high variability what supports the idea that GSyV1 population in Central Europe is more diverse than the North American ones.

#### Grapevine rupestris stem pitting-associated virus (GRSPaV)

GRSPaV is known to be the most widespread virus infecting grapevine, but to our surprise we could detect its presence by both contig blast and read bwa in only three of our samples (Supplementary Table 4). In a striking contrast, with RT-PCR using diagnostic primers amplifying a very short part of the replicase we obtained a product in 16 of our samples (Figure 2B). GRSPaV's viral RdRp has a very low proofreading activity and frequent recombination events, because the coexistence of different variants in the same plant led to the evolution of diverse variants (Morelli et al., 2011; Glasa et al., 2017). To test that if the number of virus-specific reads or coverage will increase if we use a different reference during bioinformatics analysis, we made the direct sRNA BWA using 5 distinct full GRSPaV genomes (AF057136 = NC_001948 _Ref_1_USA, AY881627_BS_Canada, KR054734_JF_China, AY881626_SG1_USA, AY368590_SY_USA) (Supplementary Table 7). According to this analysis we obtained higher than 40% coverage to different GRSPaV strains only in two additional libraries. GRSPaV is mostly spread by vegetative propagation; as a result, it is particularly difficult to eliminate by sanitation techniques and coexists with grapevine for a long time (Meng et al., 2006). Their coevolution led to gene expression changes of the host with mutual advantages, resulting in slight down-regulation of stress genes in the presence of the virus (Gambino et al., 2012). Because of these advantages it is possible that during this coevolution an acceptable balance of the virus and the host defense reaction was achieved. The presence of a GRSPaV-coded silencing suppressor with an activity to block virus-derived sRNA biogenesis can also explain these results, but must be further investigated.

To be able to analyse phylogenetical relationship of GRSPaV isolates, we amplified and sequenced a longer 3' part of their genome (Figure 2B). Hungarian variants clustered with the GRSPaV-1 and Tannat variants from the USA and Uruguay, but it is possible that sequencing more clones or full genomes would alter this phylogenetic picture (Supplementary Figure 2L).

#### Grapevine pinot gris virus (GPGV)

**Figure 3:**
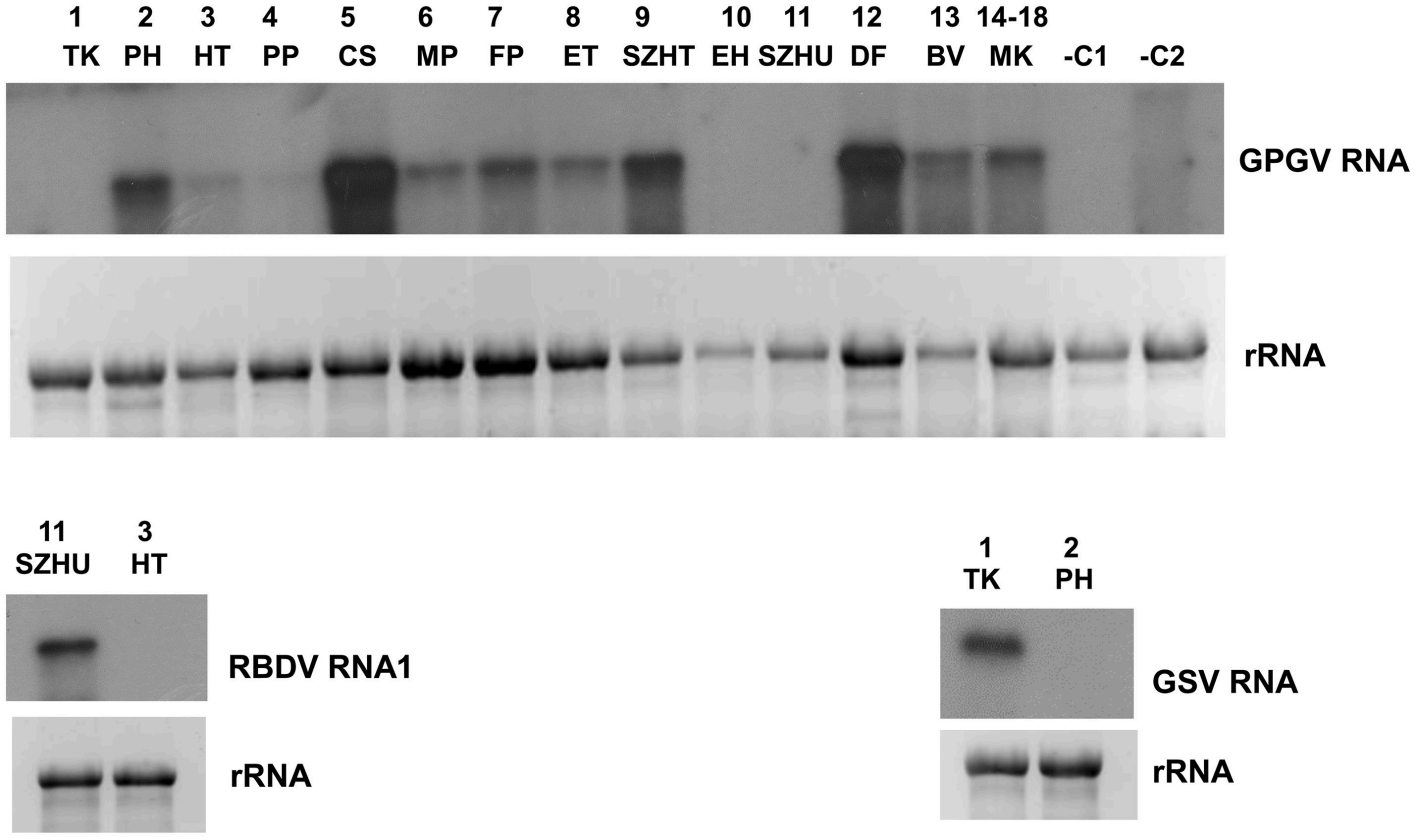
Validation of the sRNA NGS by Northern blot hybridization for A/GPGV, B/RBDV RNA1, and C/GSV. Four to five micrograms total RNA from pooled samples was separated on 1,2% agarose gel, blotted to Nytran membrane and hybridized with radioactively labeled virus specific probes. Relative gel loadings are indicated by ethidium bromide staining of ribosomal RNAs. **(A)** The presence of GPGV was investigated in all vineyards. In lane 1-13 library pools, in lane 14 plantation pool prepared from library 14-18 was used. RNA from grapevine (–C1) or *Nicotiana benthamiana* (–C2) not containing GPGV was used as negative control. **(B)**The presence of RBDV_RNA1 was investigated in 11_SZHU vineyard. RNA from library 3_HT, not containing this virus was used as negative control. **(C)**The presence of investigated in 1_TK vineyard. RNA from library 2_PH, not containing this virus was used as a negative control.

According to our survey, GPGV, never described before, seems widespread in our country: it is present in 17 libraries (Table 1). In striking contrast to the predominance of 21 nt GPGV-derived reads with both sense and antisense orientation, a 24 nt-long antisense excess was found in 1_TK and 11_SZHU (Supplementary Figure 4A). In these samples the number of virus-specific reads was very low, but we have found 2 GPGV-annotated contigs in each of them. Aligning these contigs to GPGV revealed that they were generated from the 5' part of the genome (Supplementary Table 8). In this region (155–235) the Italian Reference (NC_015782) differs from all of the other sequenced genomes and contains a stretch with exact match to *V. vinifera* shotgun sequences. Similarly to GPGV's original description (Giampetruzzi et al., 2012), our bioinformatics pipeline does not contain removal of host/grapevine-specific reads before virus diagnostics. This suggests that GPGV-identified sRNA reads in these libraries could be false positives of host origin, which what could explain their size distribution and the contradiction between contigs and the results based on direct sRNA alignment. To investigate the question why we could get a GPGV-specific RT-PCR product in 11_SZHU (Figure 2C), if the virus-derived sRNAs are false positives, extracts of individuals and different tissues were investigated by RT-PCR for the presence of the virus. The analysis showed that at this very young plantation only one plant was infected, and GPGV was present only in its young leafs and shoot tips (Supplementary Figure 3D). We proved the presence of GPGV by Northern blot in 11 vineyards (using pooled RNA for 14–18 libraries, originating from different varieties of the same plantation) (Figure 3A). In 10_EH the low amount of loaded RNA, whereas in 11_SZHU low amount of GPGV could be the reason why we couldn't get a signal. Since its first description (Giampetruzzi et al., 2012) GPGV has been reported from all over the world, including Slovakia (Glasa et al., 2014), Slovenia (Plesko et al., 2014), Croatia (Voncina et al., 2017), Serbia, Romania, and Ukraine (Bertazzon et al., 2016), i.e., almost all of Hungary's neighboring countries. According to their CP sequences our isolates showed slight variation, but grouped distantly and together with isolates of different geographical origins (Supplementary Figure 2M), which supports the possibility that GPGV spread from Eastern Europe to Italy (Bertazzon et al., 2016; Malagnini et al., 2016), and from Europe to other parts of the world (Wu and Habili, 2017). Its spread by a putative slow-moving eriophyid mite vector (Bertazzon et al., 2017) from surrounding infected plants could explain its unequal presence in our youngest (1-year-old) plantation (11_SZHU). In spite of its high prevalence, symptoms caused by GPGV are rare, and the varieties we sampled did not show symptoms connected to GPGV. Requirements for being latent or virulent strain are still elusive (Saldarelli et al., 2015); however, according to the polymorphism at the end of the MP, all isolates from our country belong to the latent group, having MPs shorter by six amino acids.

#### Raspberry bushy dwarf virus (RBDV)

Since its first description in Slovenia (Mavric et al., 2003), RBDV has been reported rarely, but also from Hungary, to infect grapevine (Plesko et al., 2012). We found the presence of RBDV in 11_SZHU, where presence of contigs, high number of normalized virus-specific and high coverage of both RNA1 and RNA2 by small RNA reads were present (Supplementary Table 4). In this very young, 1-year-old Furmint plantation we successfully validated the presence of both RNAs by RT-PCR (Figure 2C), and in case of RNA1 by Northern blot (Figure 3B). Phylogenetical analysis of the cloned part of RNA1 showed that it differs from the RBDV RNA1 sequences, which was only available from *Rubus* host (Supplementary Figure 2N upper panel). The same analysis of the MP part of RNA2 (Supplementary Figure 2N lower panel) showed that RBDV at Tokaj, in the north-eastern part of the country, is closer to the isolate originating from the Slovenian *Vitis* host than to Hungarian isolates from the same host, from the southwestern part of the country (JQ928628 and JQ928629), which strongly suggests its origin from the plantation material. Although we couldn't find RBDV-specific RT-PCR product from the 13_BV plantation pool, we tested individuals at this plantation, because RBDV-specific sRNAs were present and coverage of both RNA1 and RNA2 was about 40%. An RT-PCR product with primers amplifying RNA1 at the expected size was found in BV2 plant (Supplementary Figure 3E) and proved to be RBDV1-specific by Sanger sequencing, having 98.6% identity to the RBDV RNA1 sequence from 11_SZHU (Supplementary Figure 5). BV2 is Balafánt, an ancient Hungarian variety, which has been present in the collection for a long time, raising the question how it could be infected by this virus in the north-eastern part of the country.

In addition to viruses, we also identified a viral satellite and viroids in our vineyards.

#### Grapevine satellite virus (GSV)

Grapevine satellite virus was first described in California by deep sequencing of dsRNAs (Al Rwahnih et al., 2013) and later from Iran by the same technique (Candresse et al., 2017b). During our survey we found its presence in two of our libraries (Supplementary Table 4). In 1_TK its presence was validated by RT-PCR (Figure 2C) and also by Northern blot (Figure 3C). In 13_BV we obtained a virus-specific product by RT-PCR only when we tested individuals of this plantation (Supplementary Figure 3F). Although a helper virus of GSV has not been identified yet, its original source was infected by Vitiviruses and various GLRaVs (Al Rwahnih et al., 2013). 1_TK and 13_BV contained GVA and GLRaV1 in parallel, suggesting their helper virus function for GSV. Sequence comparison of the 927 bp cloned from GSV (HUTK) showed that is closer to the Californian Reference (NC_021480) than to the one from Iran (Supplementary Figure 2O), and it is slightly different from our other isolate (Supplementary Figure 6), making its origin more elusive.

### Viroids

#### Hop stunt viroid (HSVd)

According to our survey, almost all of our libraries contained massive amounts of HSVd-derived sRNAs. Their size distribution and polarity showed the same pattern: simultaneous presence of 21-, 22-, and 24nt-long reads originating from both strands, as it was expected on the basis of the detailed analysis of Navarro et al. (2009) (Supplementary Figure 4B). We could validate its presence in our samples by RT-PCR (Figure 2D). As HSVd sequences from different strains share high identity, we only sequenced 1 cloned PCR product originating from Tokaj. HSVd variants from the sequenced ENTAV115 Pinot noir originating from Eger (the northern part of the country) contained the Riesling variant reported from Germany (Navarro et al., 2009). Phylogenetic analysis of our isolate HUMK4 showed that although it is 95% identical to the reference genome, it is not very closely related to this Riesling strain and to another Hungarian HSVd isolate reported from Hungary (Supplementary Figure 2P).

#### Grapevine yellow speckled viroid (GYSVd1, 2)

GYSVd was found in 16 samples (Table 1). Although they are 81% identical our analysis generated results for GYSVd1 and GYSVd2, as they both have reference genomes. According to our bioinformatics analysis GYSVd1 is present in 16 of our libraries. The same analysis for GSYVd2 was not that straightforward (Supplementary Table 4). Size distribution of the GSYVd1 derived sRNAs showed again the presence of 21, 22, 24 nt-long reads, but in our samples the presence of 22 nt-long reads was not as high as it was for those originating from the HSVd or GYSVd1 published from the Pinot noir ENTAV (Navarro et al., 2009) (Supplementary Figure 4C). RT-PCR validation by GYSVd1-specific primers was successful for all of our samples (Figure 2D), but we never obtained any product if we used GYSVd2-specific primers, which would suggest that variants at Hungarian plantations are closer to GYSVd1. Sequences of three isolates, originating from different parts of the country, showed divergent variation (Supplementary Figure 2Q). Detailed analysis by multiple alignment showed hot spot regions of the genome, where its structure allows mutations, but we did not find variation at the point where variants from PN had been reported (Navarro et al., 2009) (Supplementary Figure 7); however, it might be possible to identify variants at that point by sequencing more clones.

#### Sanitary status of Hungarian plantations

In most cases we could validate the result of sRNA NGS by RT-PCR, but interestingly the number of validated viruses and viroids was sometimes higher than expected according to contig blast or read bwa (Table 1). The investigated vineyards were free of GFLV, ArMV and GLRaV2. GCMV and GLRaV3 was present at one, GVB at three, whereas GVA at 5five places. As expected, GLRaV1 and GFkV were present frequently (8 and 12 plantations, respectively), and we also found other Tymoviruses: GRGV, GAMaV, and GRVFV together with GFkV at several locations (9, 8, and 13, respectively). The most widespread infection was found for viroids (present at 14 and 13 plantations, respectively), GRSPaV and, unexpectedly, for GSyV1 and GPGV at 14, 13, and 13 locations, respectively. To our surprise, we found RBDV at two plantations, and we first described GSV in our country.

Most of the vineyards were infected with different viral pathogens in parallel, and we couldn't find any correlation between the age of the plantation and the number of viruses and viroids present. However, the most infected plantation 12_DF is about 100 years old (infected by 12 viral pathogens), the youngest, 1-year-old plantation 11_SZHU contained 9 viral pathogens, which suggests that the infection is usually brought about by the use of infected propagation material. Moreover, the sanitary status of the MK plantation at Tokaj (14-18_MK), containing different varieties, differed from one variety to another. The least infected variety was the rootstock variety Teleki-Kober 125 (infected only with GPGV and slightly with 1 viroid), whereas the most infected varieties were the frequently used Furmint and Hárslevelu clones (infected with eight viruses). This significant difference in viral status between the rows of the same plantation reduces the possibility of an on-site infection from the neighborhood by viral vectors.

## Conclusions

Combination of next generation sequencing, bioinformatics, and molecular biology techniques provide us a powerful new high-throughput diagnostic tool to monitor grapevine plantations and get a deep insight into the virus infection status of Hungarian vineyards.

During our comprehensive survey, beside routinely tested viruses, presence of viruses never described in Hungary before, was revealed. Phylogenetic analysis of partial sequence of the virus isolates suggests that infection usually happened through the infected propagation material, highlighting the importance of virus diagnostic surveys by more sensitive methods than the routinely used ones.

Traditional diagnostic methods can only answer the question if a particular pathogen is present in the sample or not. As a contrast sRNA NGS, as a metagenomics technique, seems to be a powerful new high-throughput diagnostic tool. Using this approach, presence of grapevine-infecting regulated viruses can be reliable determined. Randomly collected, pooled samples, instead of samples from selected symptomatic plants, offer an alternative, unbiased way to reveal the presence of viral pathogens in the vineyards, but uneven presence of viruses in the sampled plants could lower the concentration of the virus in the pooled library, which could lead to false negative result. However our results showed that false negative results originating from the presence of SNPs in the genome of the isolates, at the place of the genome where diagnostic primers were designed, can be avoided.

Surveys by high-throughput methods, like sRNA NGS, will continuously provide us information about the presence and importance of different viral pathogens and can support the development of new sensitive tests for their routine diagnostics. Moreover, NGS has shown to be superior, both in reliability and speed, to biological indexing in grapevine (Al Rwahnih et al., 2015). NGS could lead to frequent discovery of new viruses (Massart et al., 2017), but beside that it can become a diagnostic method itself, in the future. Although we show that sRNA NGS-based metagenomics is one of the most reliable virus diagnostic methods, our work emphasizes that before becoming a diagnostic tool, to be adopted in the certification protocols, not only drop in the sequencing cost, but standardization and improvement of the bioinformatics pipeline is highly needed. Regular check of stock collections of varieties and rootstock plantations with this new sensitive method would prevent false negative diagnostics of the propagating material and could help maintaining the health of the vineyards in the future.

## Author contributions

NC: sRNA library preparation, molecular biology work, evaluation of data; JM, ES, and LeK: Bioinformatics analysis; ED: Molecular biology work; IB: sRNA library preparation; GS, LaK, GB, and GT: Evaluation of data, manuscript preparation; TD and JB: Experimental design, evaluation of data, manuscript preparation; EV: Experimental design, evaluation of data, writing of the manuscript.

### Conflict of interest statement

The authors declare that the research was conducted in the absence of any commercial or financial relationships that could be construed as a potential conflict of interest.
