# Supplementary material for: NGS of Virus-Derived Small RNAs as a Diagnostic Method Used to Determine Viromes of Hungarian Vineyards
Source: Front Microbiol. 2018 Feb 6;9:122. doi: 10.3389/fmicb.2018.00122 (PMC5808220; doi:10.3389/fmicb.2018.00122)
Supplement: Supplementary file 2 [file Image1.PDF]

Supplementary Material

NGS of virus derived small RNAs as a diagnostics method used to determine viromes of Hungarian vineyards

Nikoletta Czotter, Janos Molnar, Emese Szabó, Emese Demian, Levente Kontra, Ivett Baksa, Gyorgy Szittya, Laszlo Kocsis, Tamas Deak, Gyorgy Bisztray , Gabor E. Tusnady, Jozsef Burgyan and Eva Varallyay*

*** Correspondence:** Eva Varallyay: varallyay.eva@abc.naik.hu

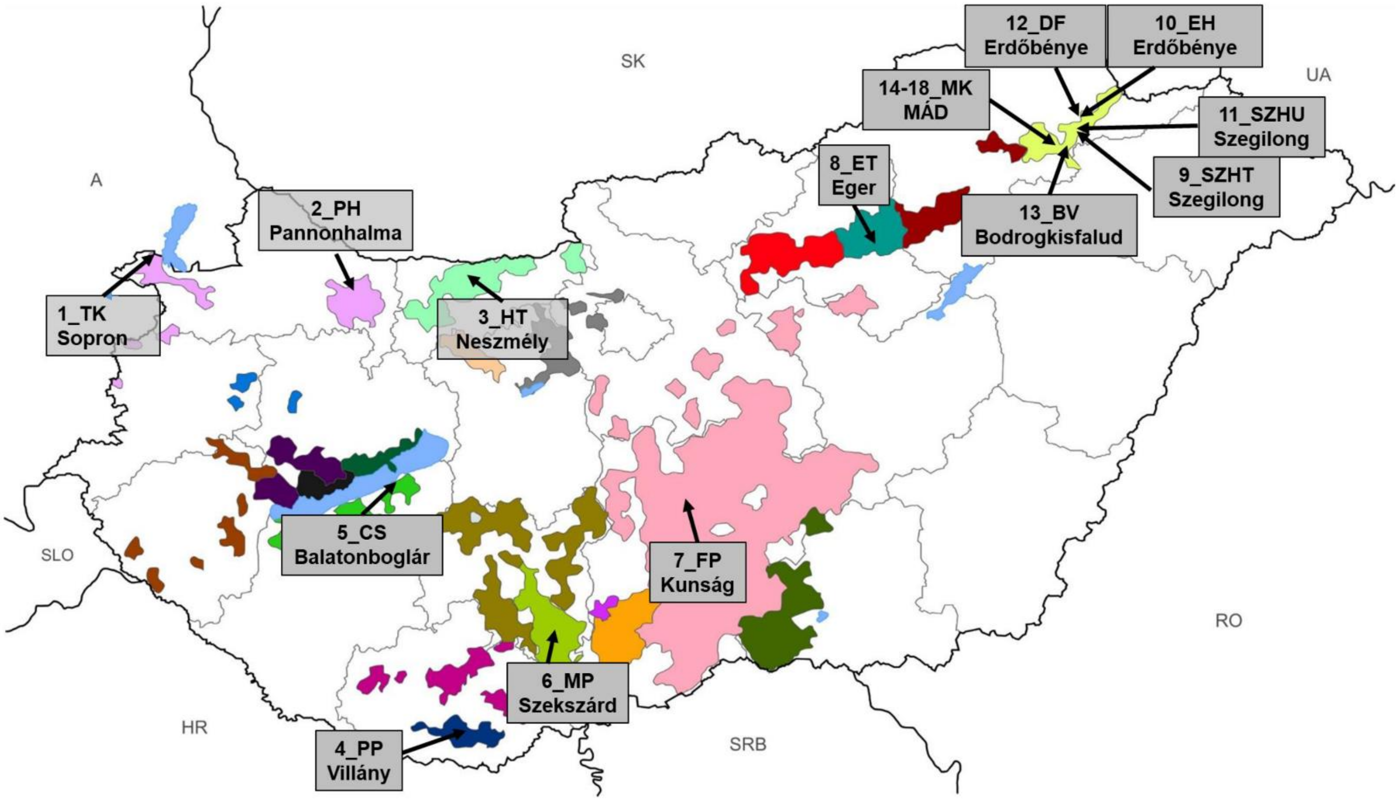

Supplementary Figure 1. Location of sampled vineyards with the number of the small RNA library.

A

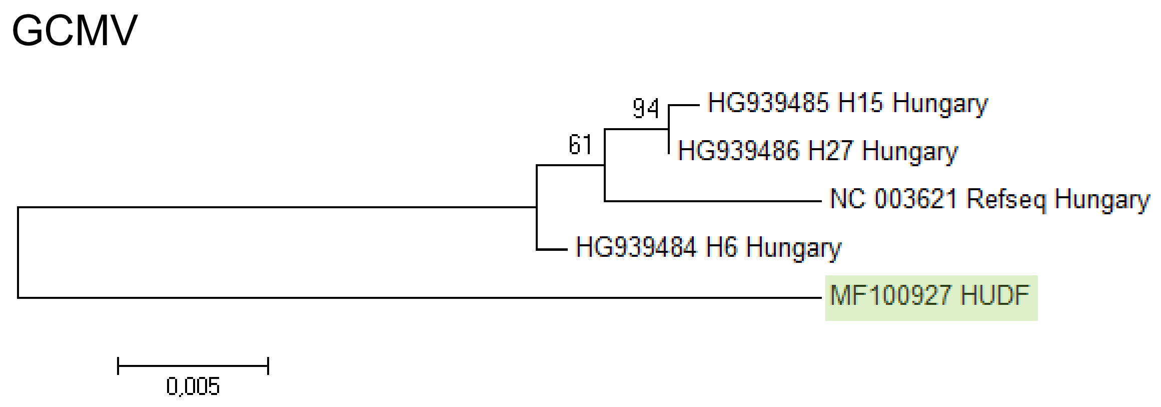
GCMV
HG939485 H15 Hungary
HG939486 H27 Hungary
NC 003621 Refseq Hungary
HG939484 H6 Hungary
MF100927 HUDF
94
61
0,005

B

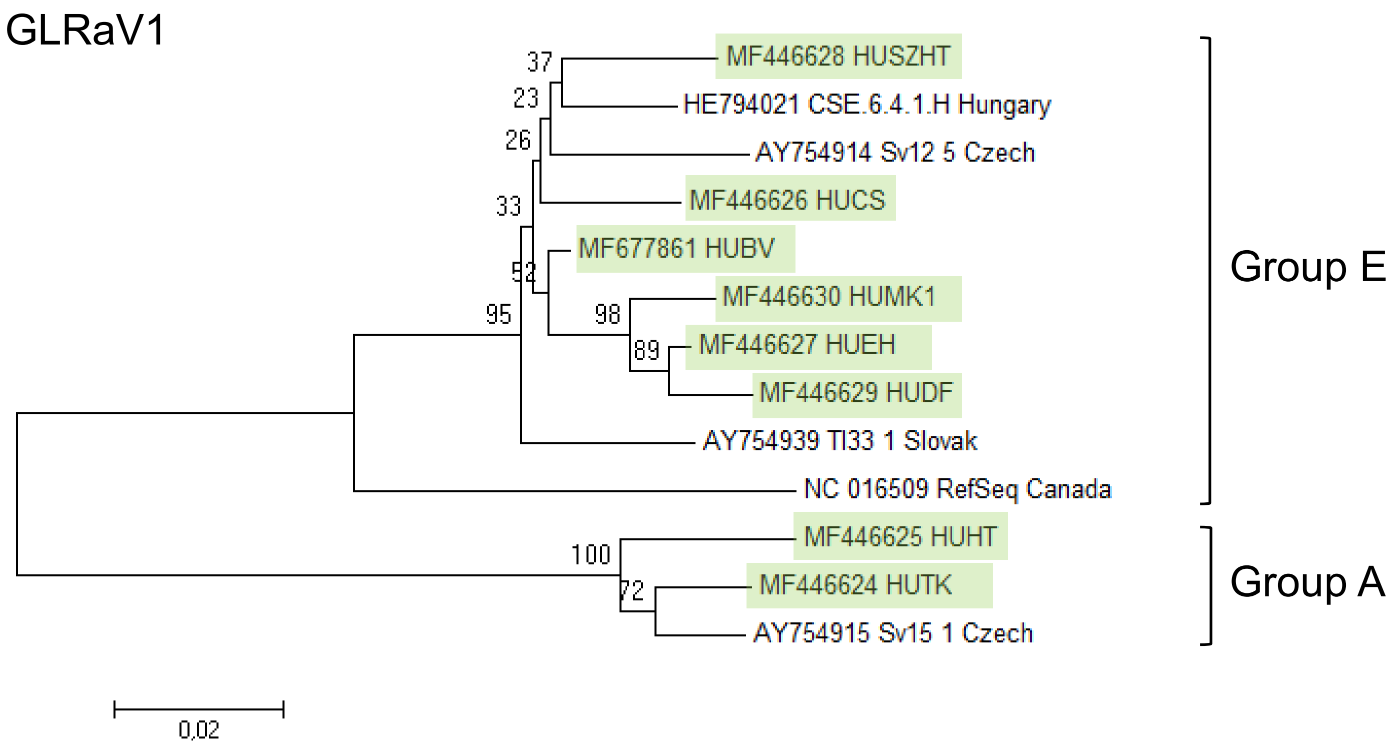
GLRaV1
MF446628 HUSZHT
HE794021 CSE.6.4.1.H Hungary
AY754914 Sv12 5 Czech
MF446626 HUCS
MF677861 HUBV
MF446630 HUMK1
MF446627 HUEH
MF446629 HUDF
AY754939 TI33 1 Slovak
NC 016509 RefSeq Canada
MF446625 HUHT
MF446624 HUTK
AY754915 Sv15 1 Czech
37
23
26
33
52
95
98
89
100
72
Group E
Group A
0,02

C

GLRaV3 - CP

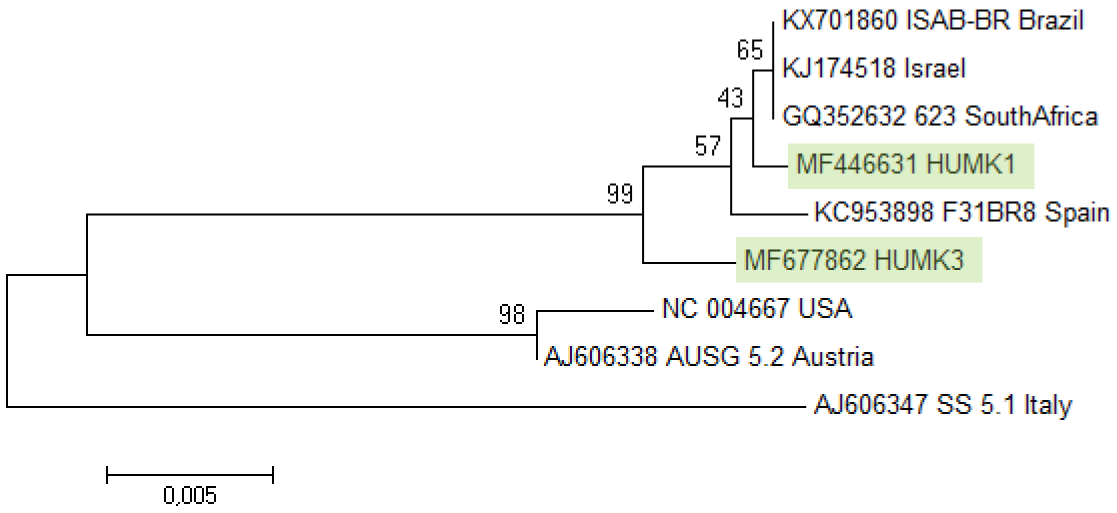

D

GLRaV3 – HSP70

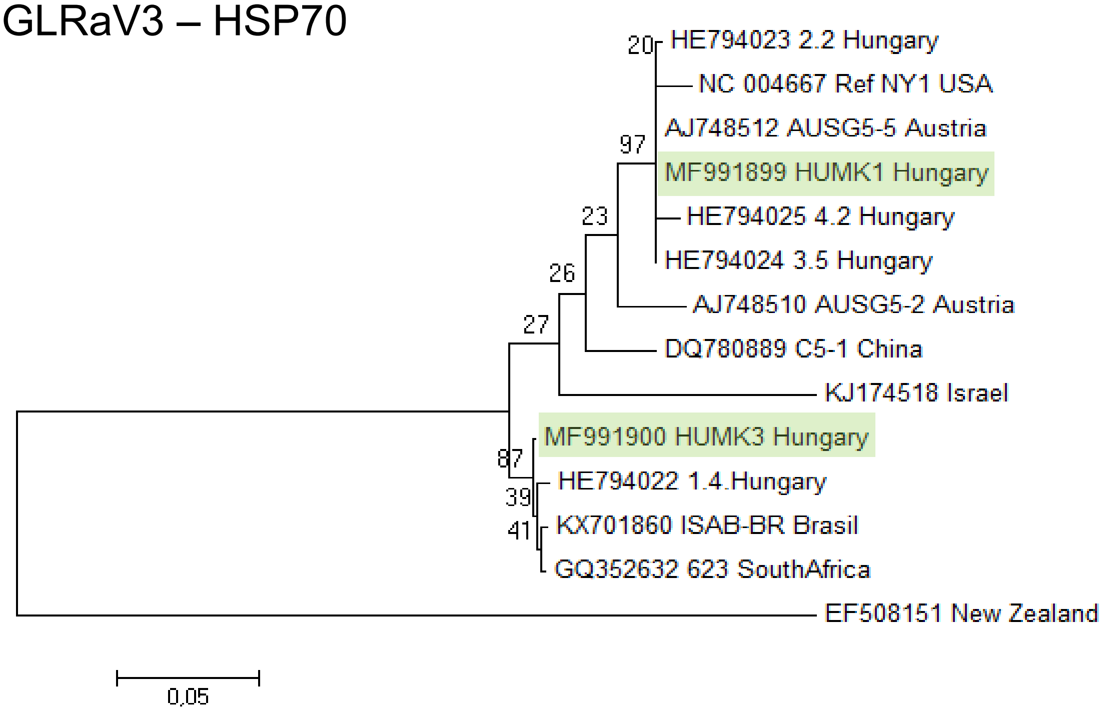

E

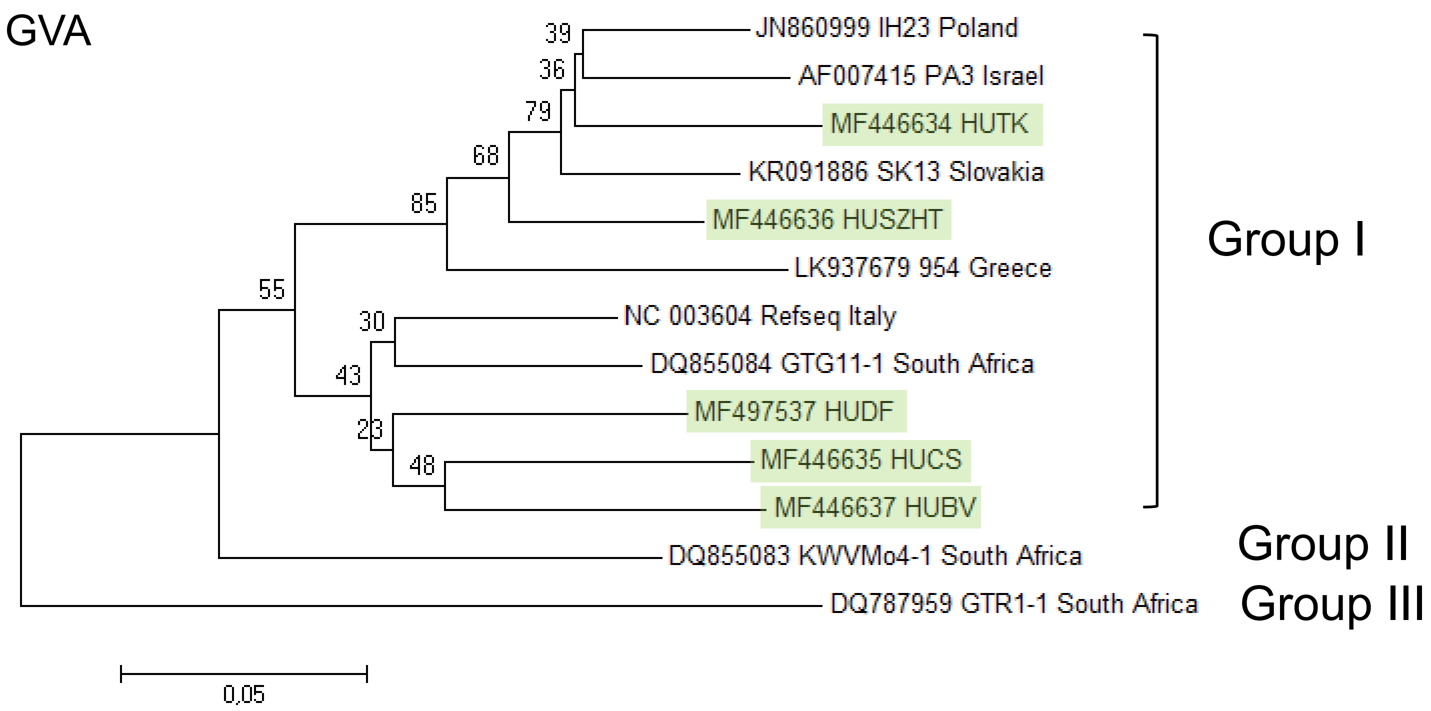
GVA
JN860999 IH23 Poland
AF007415 PA3 Israel
MF446634 HUTK
KR091886 SK13 Slovakia
MF446636 HUSZHT
LK937679 954 Greece
NC 003604 Refseq Italy
DQ855084 GTG11-1 South Africa
MF497537 HUDF
MF446635 HUCS
MF446637 HUBV
DQ855083 KWVMo4-1 South Africa
DQ787959 GTR1-1 South Africa
Group I
Group II
Group III
39
36
79
68
85
55
30
43
23
48
0,05

F

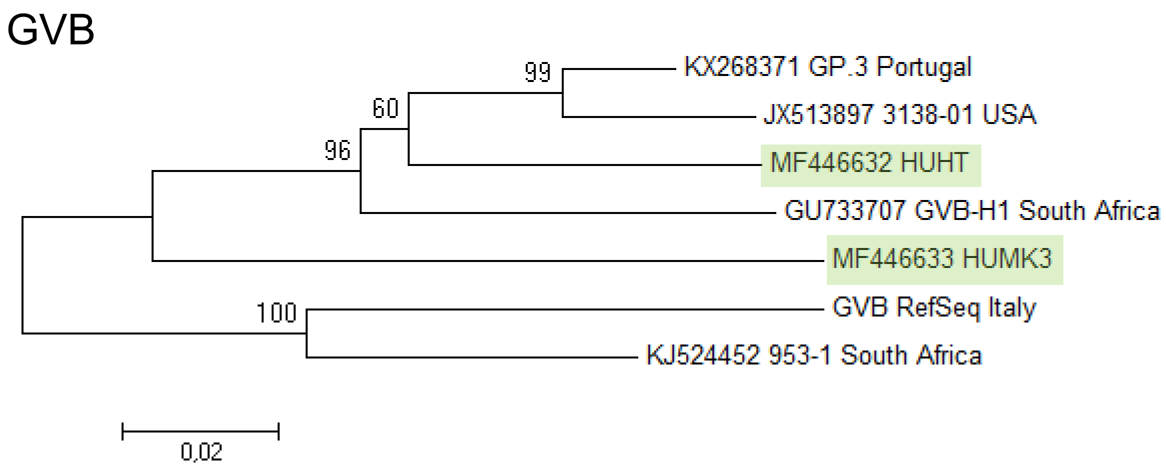
GVB
KX268371 GP.3 Portugal
JX513897 3138-01 USA
MF446632 HUHT
GU733707 GVB-H1 South Africa
MF446633 HUMK3
GVB RefSeq Italy
KJ524452 953-1 South Africa
99
60
96
100
0,02

G

GFkV

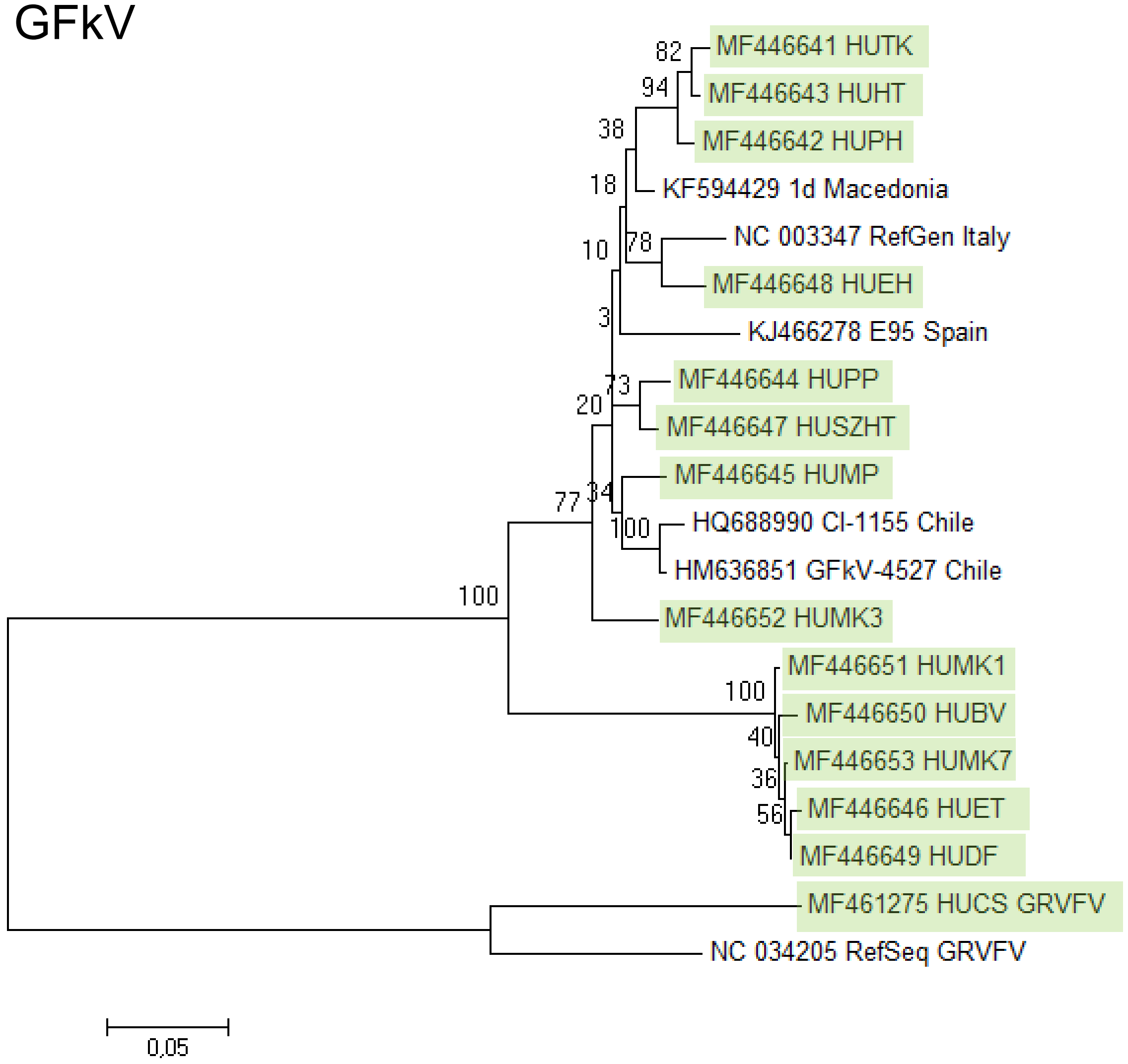
82
94
38
18
10
78
3
73
20
34
77
100
100
100
40
36
56
MF446641 HUTK
MF446643 HUHT
MF446642 HUPH
KF594429 1d Macedonia
NC 003347 RefGen Italy
MF446648 HUEH
KJ466278 E95 Spain
MF446644 HUPP
MF446647 HUSZHT
MF446645 HUMP
HQ688990 CI-1155 Chile
HM636851 GFkV-4527 Chile
MF446652 HUMK3
MF446651 HUMK1
MF446650 HUBV
MF446653 HUMK7
MF446646 HUET
MF446649 HUDF
MF461275 HUCS GRVFV
NC 034205 RefSeq GRVFV
0,05

H

GRGV

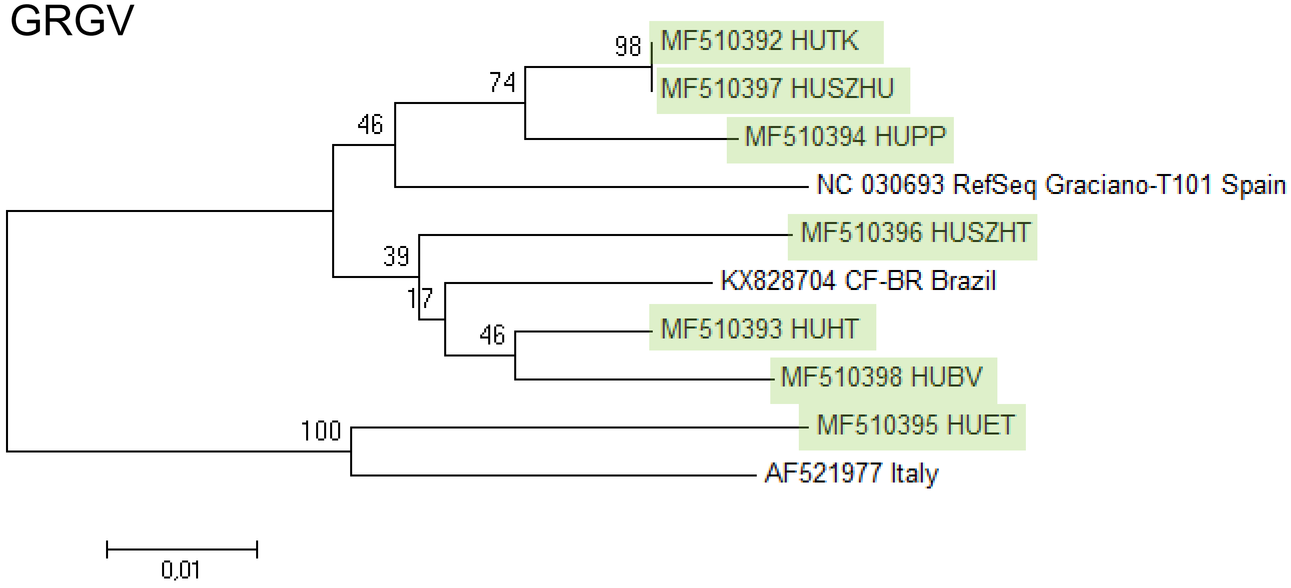

I

GAMaV

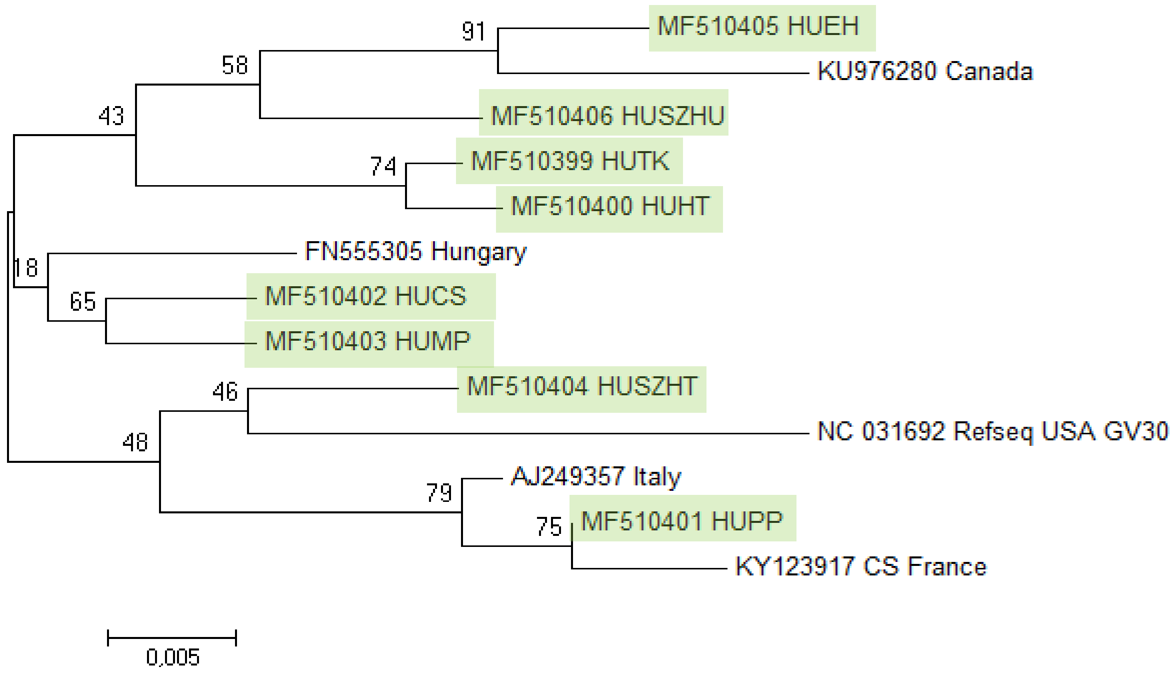

J

GRVFV

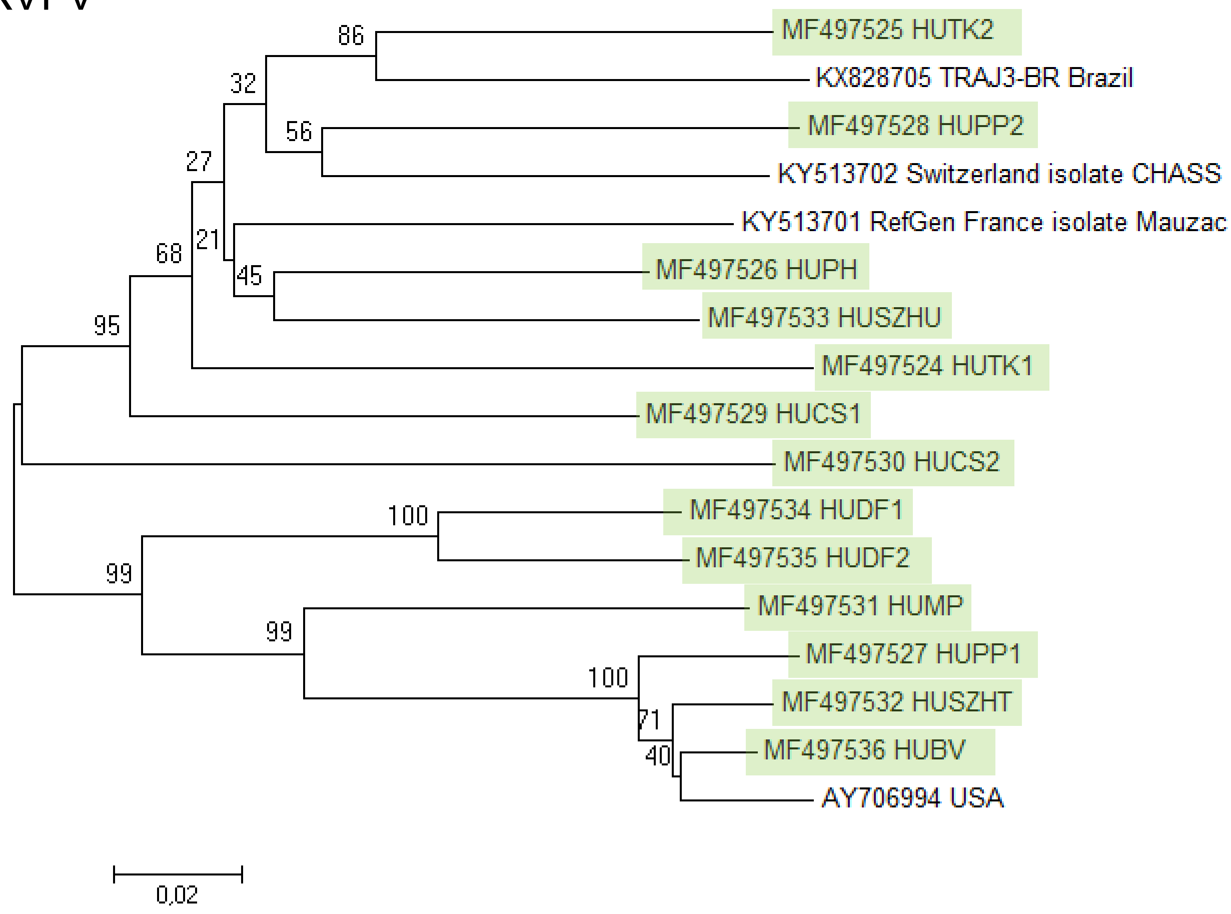
86
32
56
27
21
45
68
95
MF497525 HUTK2
KX828705 TRAJ3-BR Brazil
MF497528 HUPP2
KY513702 Switzerland isolate CHASS
KY513701 RefGen France isolate Mauzac
MF497526 HUPH
MF497533 HUSZHU
MF497524 HUTK1
MF497529 HUCS1
MF497530 HUCS2
100
99
99
100
71
40
MF497534 HUDF1
MF497535 HUDF2
MF497531 HUMP
MF497527 HUPP1
MF497532 HUSZHT
MF497536 HUBV
AY706994 USA
0,02

K

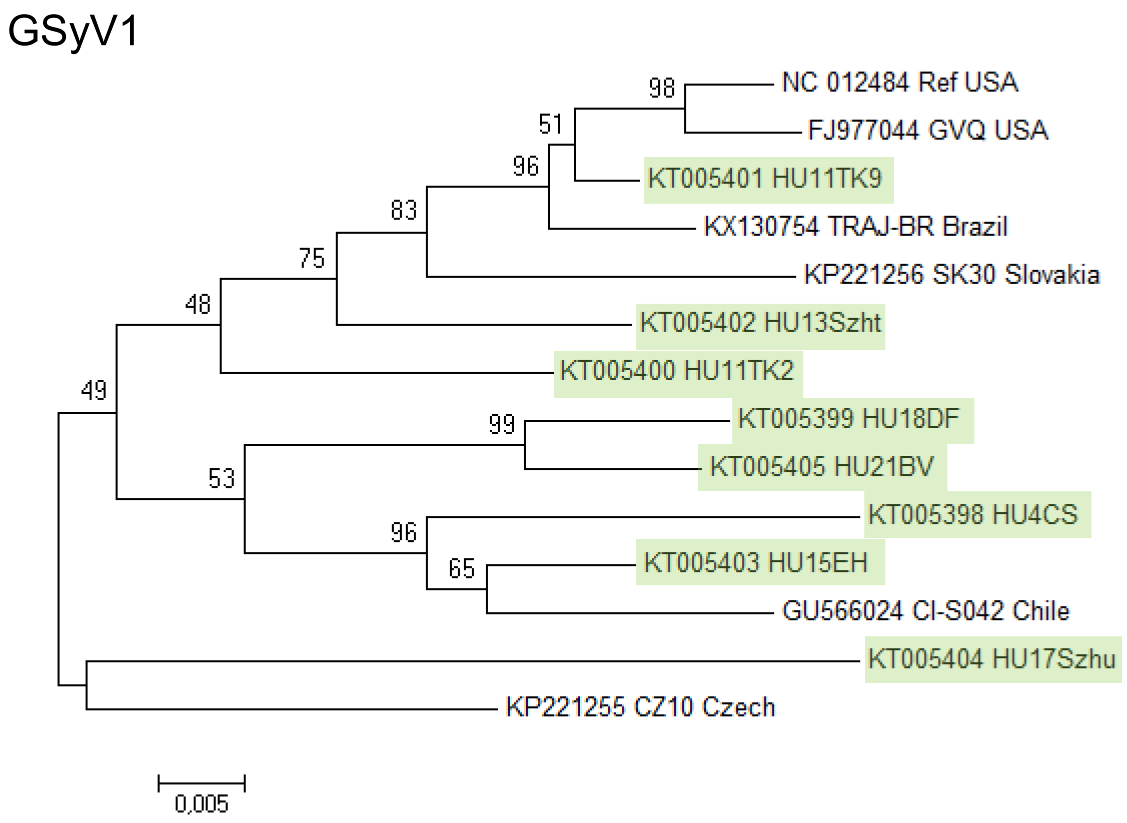
GSyV1
98
51
96
83
75
48
49
99
53
96
65
NC 012484 Ref USA
FJ977044 GVQ USA
KT005401 HU11TK9
KX130754 TRAJ-BR Brazil
KP221256 SK30 Slovakia
KT005402 HU13Szht
KT005400 HU11TK2
KT005399 HU18DF
KT005405 HU21BV
KT005398 HU4CS
KT005403 HU15EH
GU566024 CI-S042 Chile
KT005404 HU17Szhu
KP221255 CZ10 Czech
0,005

L

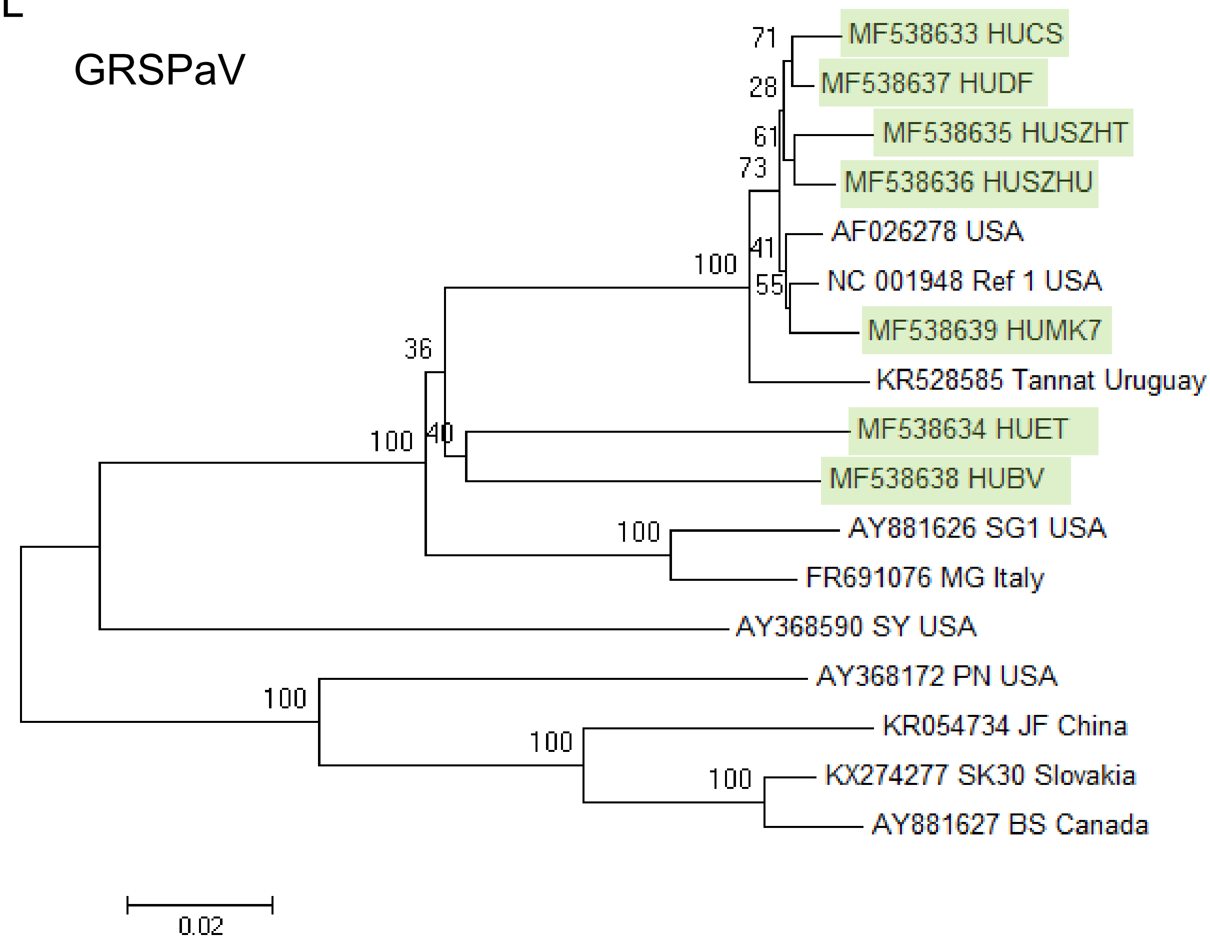
GRSPaV
MF538633 HUCS
MF538637 HUDF
MF538635 HUSZHT
MF538636 HUSZHU
AF026278 USA
NC 001948 Ref 1 USA
MF538639 HUMK7
KR528585 Tannat Uruguay
MF538634 HUET
MF538638 HUBV
AY881626 SG1 USA
FR691076 MG Italy
AY368590 SY USA
AY368172 PN USA
KR054734 JF China
KX274277 SK30 Slovakia
AY881627 BS Canada
71
28
61
73
41
55
100
36
100
40
100
100
100
100
0.02

M

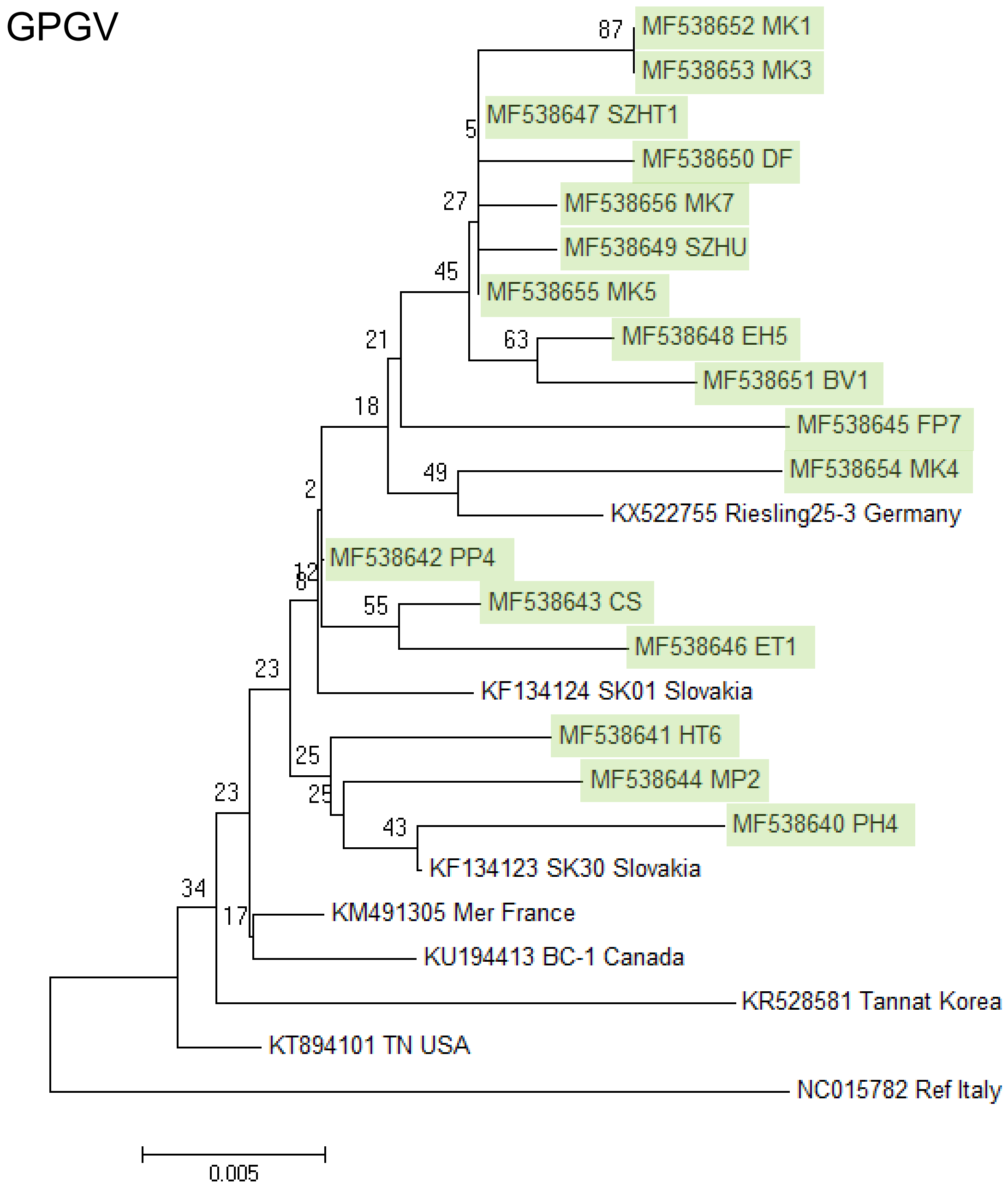
GPGV
87
MF538652 MK1
MF538653 MK3
5
MF538647 SZHT1
MF538650 DF
27
MF538656 MK7
MF538649 SZHU
45
MF538655 MK5
21
63
MF538648 EH5
MF538651 BV1
18
MF538645 FP7
49
MF538654 MK4
KX522755 Riesling25-3 Germany
2
MF538642 PP4
12
8
55
MF538643 CS
MF538646 ET1
23
KF134124 SK01 Slovakia
MF538641 HT6
25
MF538644 MP2
25
23
43
MF538640 PH4
KF134123 SK30 Slovakia
34
17
KM491305 Mer France
KU194413 BC-1 Canada
KR528581 Tannat Korea
KT894101 TN USA
NC015782 Ref Italy
0.005

N

RBDV_RNA1

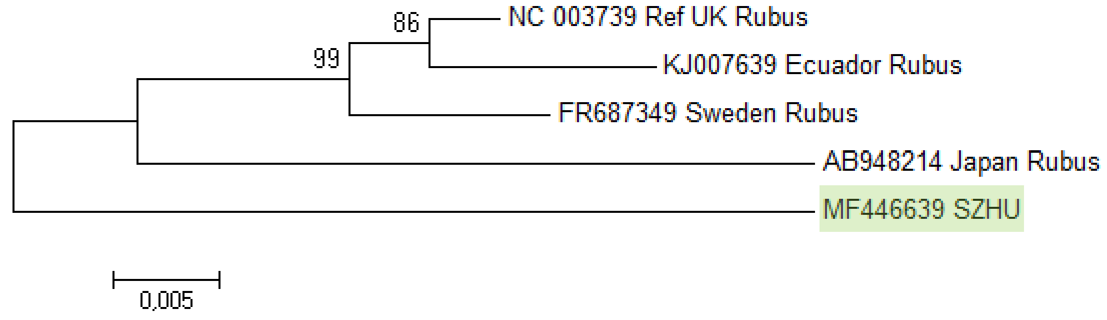

RBDV_RNA2

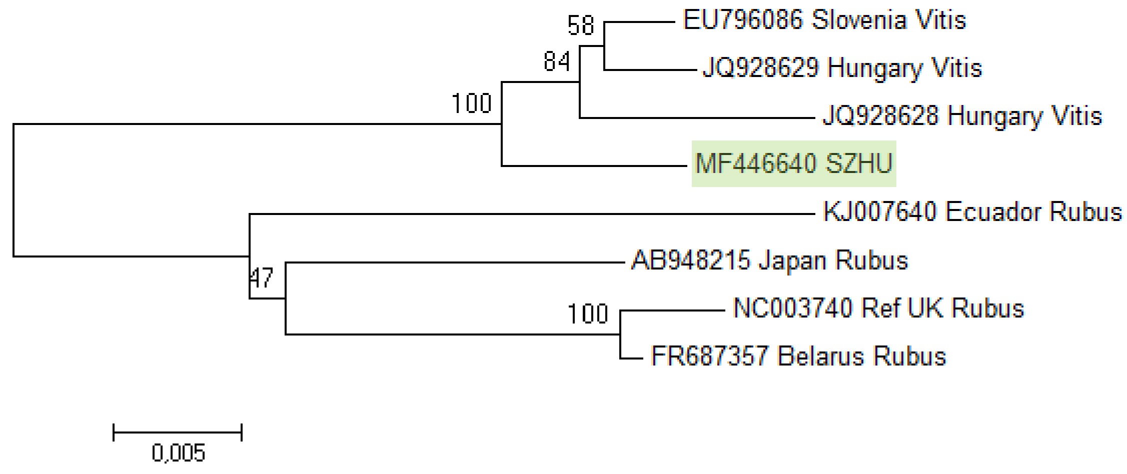

O

GSV

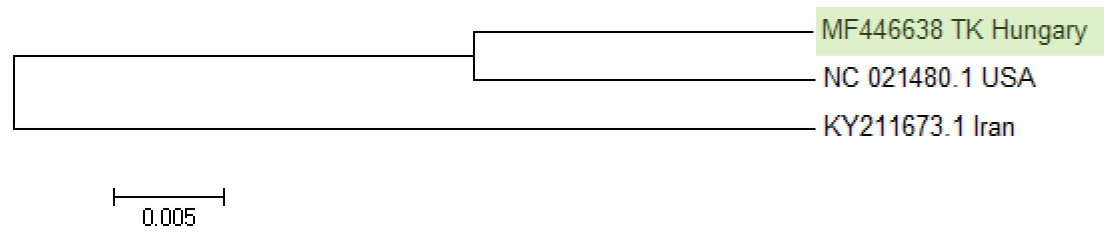

P

HSVd

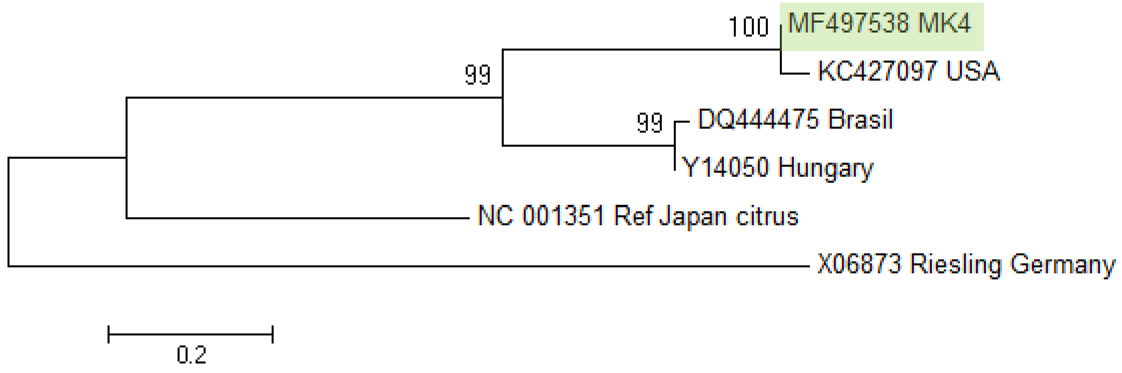

Q

GYSVd

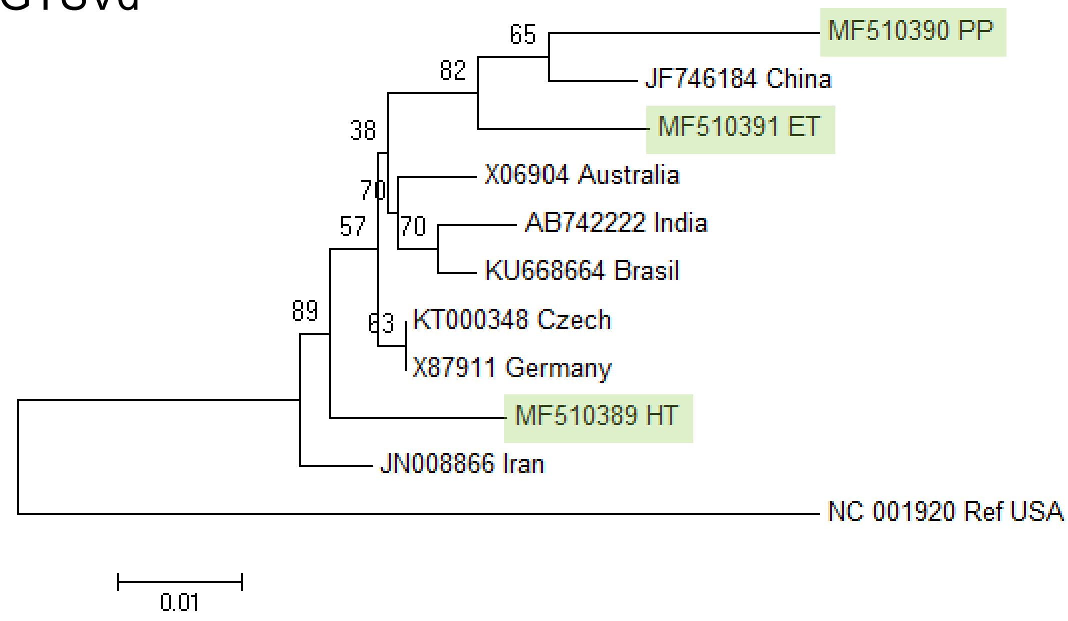

Supplementary Figure 2.
Phylogenetic relationships based on partial sequences of A/GCMV, B/GLRaV1, C/GLRaV3 coat protein, D/GLRaV3 HSP70, E/GVA, F/GVB, G/GFKV, H/GRGV, I/GAMaV, J/GRVFV, K/GSyV1, L/GRSPaV, M/GPGV, N/RDBV, O/GSV, P/HSVd, Q/GYSVd isolates with reference genomes and other known isolates of NCBI GenBank. The green boxes indicate a virus or a viroid sequenced in this study, along with their respective accession numbers. Phylogenetic trees was constructed by MEGA v6 using neighbor-joining algorithm. Bootstrap values >70% (1000 bootstrap replicates) are given at the branch nodes.

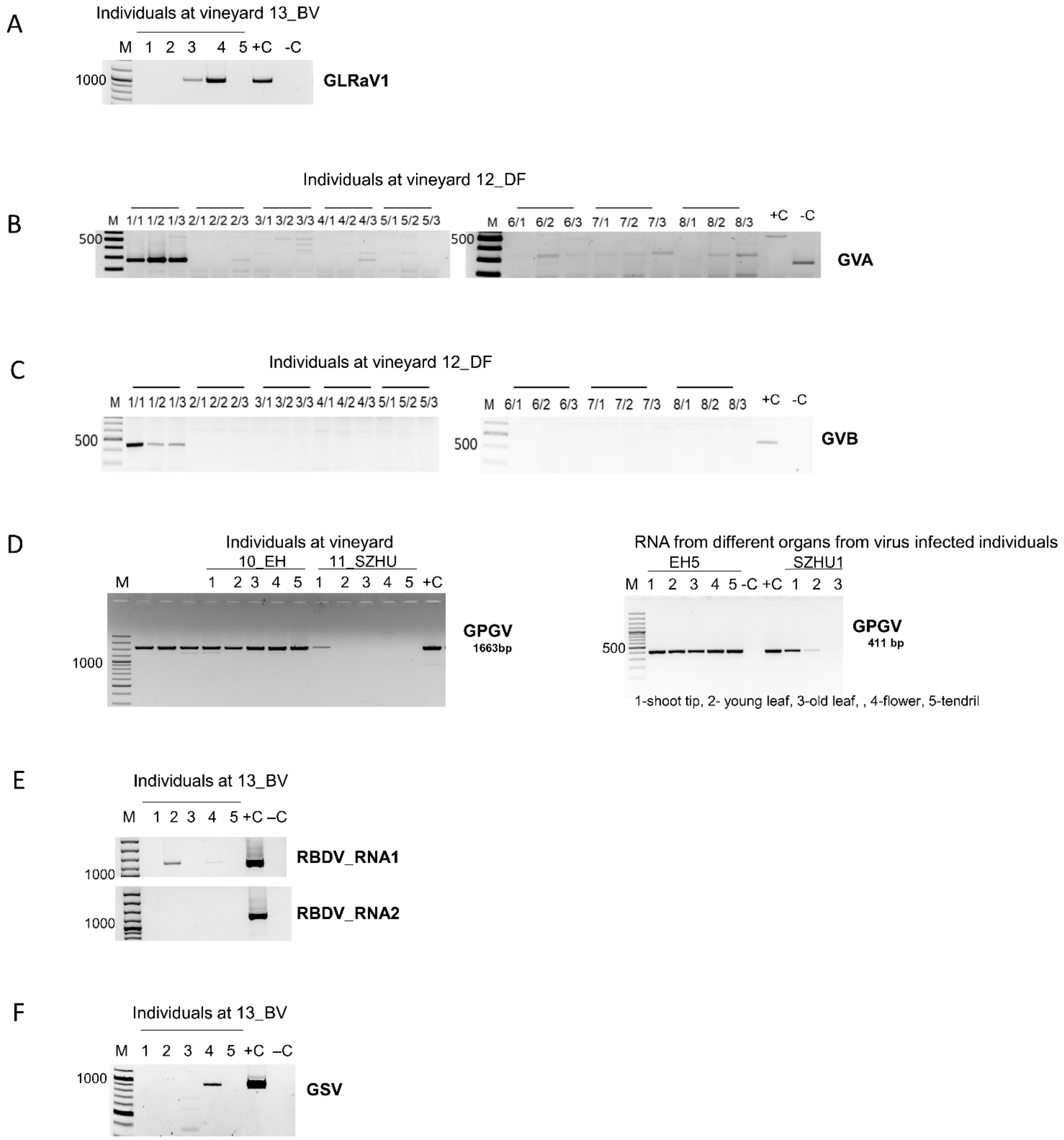

Supplementary Figure 3.
RT-PCR validation of sRNA NGS testing individuals of the particular vineyards for the presence of A/GLRaV1, B/GVA, C/GVB, D/GPGV, E/RBDV and F/GSV. cDNA was synthetized from pooled RNA extracts representing individuals, or RNA extracted from different organs of the same plant using random primer and used as templates for PCR reactions. PCR products were analysed by agarose gel electrophoresis. (M): GenRuler 100bp+; (+C): cDNA containing the tested virus was used as positive, or (-C): water as negative control.

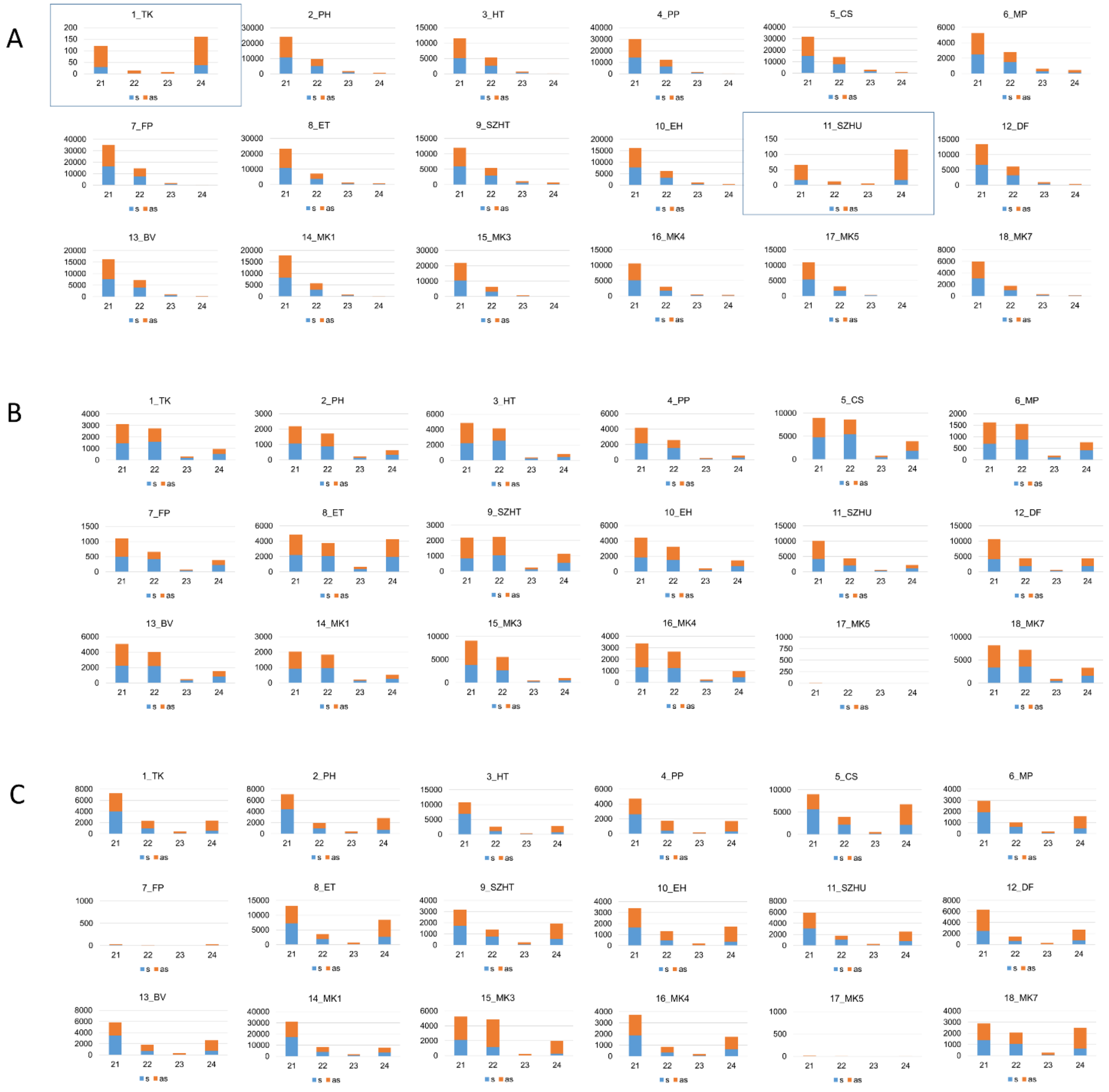

Supplementary Figure 4

Size distribution of the specific virus derived sRNAs with their polarity – orange:negative strand, blue-sense strand origin A/ /GPGV, B/HSVd, C/GYSVd1

```
NC003739_Ref_UK_Rubus      AAGGTGACGGACCCAGTCTGTGTTGCCTTATCCCCAATTCAAAAATACAAACATATGATT
RBDV_RNA1_BV2              ------ACAGACCCAGTCTGTGTTGCCTTATCCCCAATGCAAAAATATAAACATATGATT
MF446639_SZHU              AAGGTAACAGACCCAGTCTGTGTTGCCTTATCCCCAATGCAAAAATATAAACATATGATT
                                 ** ***************************** ******** ************

NC003739_Ref_UK_Rubus      AAGAGGGATGTCAAATTCAATTTGACTGATGGTGCTCAAAGTGAGTATACTAAAGCTGCC
RBDV_RNA1_BV2              AAGAGGGACGTCAAATTTAATTTGACTGATGGCGCTCAAAGTGAATATACTAAAGCTGCC
MF446639_SZHU              AAGAGGGACGTCAAATTTAATTTGACTGATGGCGCTCAAAGTGAATATACTAAAGCTGCC
                           ******** ******** ************** *********** ***************

NC003739_Ref_UK_Rubus      ACCATTACTTATCATCAACCAGAGATAACTCAGGTTGCTACTGCCATTTTTGGTCAGTTT
RBDV_RNA1_BV2              ACCATTACTTATCATCAACCAGAGATAACTCAGGTTGCTACAGCCATTTTCGGTCAGTTT
MF446639_SZHU              ACCATTACTTATCATCAACCAGAGATAACTCAGGTTGCTACAGCCATTTTCGGTCAGTTT
                           ***************************************** ******** *********

NC003739_Ref_UK_Rubus      AAAACTAGACTGTTGGCTTGTAGAAATAAGTTCTTAAATATACCTCTTGAACATGATAAT
RBDV_RNA1_BV2              AAAACTAGACTGTTGGCTTGTAGAAATAAGTTTTTAAATATACCTCTTGAACATGATAAT
MF446639_SZHU              AAAACTAGACTGTTGGCTTGTAGAAATAAGTTCTTAAATATACCTCTTGAACATGATAAT
                           ******************************** ***************************

NC003739_Ref_UK_Rubus      GATTTGAGCGGATATTTAACCAAATATCATTTGGGGAGTGAAAATAACACTTTCACTGAA
RBDV_RNA1_BV2              GATTTAAGCGGATATTTAACCAAATATCATTTGGGGAGTGAAAATAACACTTTCACTGAA
MF446639_SZHU              GATTTAAGCGGGTATTTAACCAAATATCATTTGGGGAGTGAAAATGACACTTTCACTGAA
                           ***** ***** ********************************* **************

NC003739_Ref_UK_Rubus      ATTGACTTTTCTAAATTCGATAAAAGTCAAGGGGAAATCCATCAACTCATTCAGGATTTA
RBDV_RNA1_BV2              ATTGATTTTTCCAAATTCGACAAAAGTCAAGGGGAAATCCATCAACTTATTCAGGATTTA
MF446639_SZHU              ATTGATTTTTCCAAATTCGACAAAAGTCAAGGGGAAATCCATCAACTTATTCAGGATTTA
                           ***** ***** ******** ************************** ************

NC003739_Ref_UK_Rubus      ATCCTGATAAAGTTCGGTTGTGATCCCGAGTTTGTAGCCTTATGGTCTACCGCCCACAGA
RBDV_RNA1_BV2              ATCCTGATAAAATTCGGTTGTGATCCCGAGTTTGTAGCCTTATGGTCCACCGCTCACAGA
MF446639_SZHU              ATCCTGATAAAATTCGGTTGTGATCCCGAGTTTGTAGCCTTATGGTCCACCGCTCACAGA
                           *********** *********************************** ***** ******

NC003739_Ref_UK_Rubus      AGTTCTTCTATTTTCGATCAAAACGTTGGAATTGGTTTTAAAACTGATTTCCAAAGAAGA
RBDV_RNA1_BV2              TGTTCTTCTATTTTCGATCAGAACGTTGGAATTGGTTTTAAAACTGACTTCCAAAGAAGA
MF446639_SZHU              TGTTCTTCTATTTTCGATCAGAACGTTGGAATTGGTTTTAAAACTGACTTCCAAAGAAGA
                            ******************* ************************** ************

NC003739_Ref_UK_Rubus      ACGGGCGATGCTTTTACTTTTCTGGGAAACTCTTTGGTAACTGCCGCCATGCTGGCGTTT
RBDV_RNA1_BV2              ACGGGCGATGCTTTCACTTTTCTGGGAAACTCTTTGGTAACTGCCGCCATGCTGGCGTTT
MF446639_SZHU              ACGGGCGATGCTTTCACTTTTCTGGGAAACTCTTTGGTAACTGCCGCCATGCTGGCGTTT
                           ************** *********************************************

NC003739_Ref_UK_Rubus      GTCATCAGTGACCCAGATAGGGAGAAGATTAGGTACATGTTGGTGGGTGGGGACGATTCT
RBDV_RNA1_BV2              GTCATCAGTGACTCGGATAGGGAGAAGATTAGGTACATGTTGGTAGGTGGGGACGATTCC
MF446639_SZHU              ATCATCAGTGACTCAGACAGGGAGAAGATTAGGTACATGTTGGTAGGTGGGGACGATTCC
                            *********** * ** ************************** ************** 

NC003739_Ref_UK_Rubus      TTGATCTGCTCCTACGGTCCAATACAAGTACCTTTGGAACCATTGGGTGACATATTTAAT
RBDV_RNA1_BV2              TTGATCTGTTCATACGGGCCAATACAAGTACCTTTAGAACCATTATGCGACATATTTAAT
MF446639_SZHU              TTGATCTGTTCCTACGGGCCAATACACGTACCTTTAGAACCATTATGCGACATATTTAAT
                           ******** ** ***** ******** ******** ********  * ************

NC003739_Ref_UK_Rubus      ATGTCTTGCAAGTTGGTACAACCAGCTTGTCCTTATTTCGCGTCTCGCTACTTGATTAGG
RBDV_RNA1_BV2              ATGTCATGCAAGTTGGTACAACCAGCTTGCCCTTATTTCGCGTCTCGCTATTTAATTAGG
MF446639_SZHU              ATGTCATGCAAGTTGGTACAACCAGCTTGCCCTTATTTCGCGTCTCGCTATTTAATTAGG
                           ***** *********************** ******************** ** ******

NC003739_Ref_UK_Rubus      AGAGGTGACGAAATTTTGTGTGTTCCCGACCCCTACAAACTTTTGGTGAAGTTGGGGAGG
RBDV_RNA1_BV2              AGAGGTGACGAAATTTTATGTGTTCCCGACCCCTATAAACTTTTAGTAAAATTGGGGAGG
MF446639_SZHU              AGAGGTGACGAAATTTTATGTGTTCCCGACCCCTATAAACTTTTAGTAAAATTGGGGAGG
                           ***************** ***************** ******** ** ** *********

NC003739_Ref_UK_Rubus      AAAGACGTCCCGGACAATCAAGCATCATTATGCGAGATACGTACCGGATTGGCAGATAGT
RBDV_RNA1_BV2              AAAGACATTCCGGACAATCAAGCATCATTATGCGAGATACGTACCGGATTGGCAGATAGT
MF446639_SZHU              AAAGACATTCCGGACAATCAAGCATCATTATGCGAGATACGTACCGGATTGGCAGATAGT
                           ****** * ***************************************************
```

```
NC003739_Ref_UK_Rubus      GCCAAATATATCTTTGATGATATTGTGAAACAGAAGTTGGCTATTCTTGTACAGGTGCGC
RBDV_RNA1_BV2              GCCAAATATATCTTTGACGATATTGTGAAACAAAAATTGGCTATTCTTGTACAAGTGCGC
MF446639_SZHU              GCCAAATATATCTTTGACGATATTGTGAAGCAAAAATTGGCTATTCTTGTACAAGTGCGC
                           ***************** *********** ** ** ***************** ******

NC003739_Ref_UK_Rubus      TATAATAAAGCTGCACCTAGTTTATATGATGCCCTTTGCACTGTGCATTGGGCATTATCT
RBDV_RNA1_BV2              TATAATAAAGCTGCACCCAGTTTATATGATGCCCTTTGTACTGTACATTGGGCATTATCT
MF446639_SZHU              TATAATAAAGCTGCACCCAGTTTATATGATGCCCTTTGTACTGTACATTGGGCATTATCT
                           ***************** ******************** ***** ***************
```

```
    1: NC003739_Ref_UK_Rubus  100.00   94.34   94.34
    2: RBDV_RNA1_BV2           94.34  100.00   98.66
    3: MF446639_SZHU           94.34   98.66  100.00
```

Supplementary Figure 5

Multiple sequence alignment of RBDV RNA1 of the reference genome and RBDV in 11_SZHU and 13_BV using Clustal Omega program. NC003739_Ref_UK_Rubus: reference genome of RBDV_RNA1; MF446639_SZHU: RBDV RNA1 sequence from 11_SZHU vineyard plantation, isolate deposited to NCBI Genbank database; RBDV_RNA1_BV2: RBDV_RNA1 sequence from BV2 individual of BV plantation. Green boxes indicate SNPs present in MF446639_SZHU or in RBDV_RNA1_BV2. Grey boxes indicate SNPs present only in the reference genome.

```
MF446638_TK           TCTTACAATCCCTAGCGCTGGACACACCCCTCTTCTTACTGTTCAGTTGCTATCTTCTTC
KY211673_Iran         -----CAATCCCTAGCGCTGGACATACCCCTCTTCTTTCTGTTCAGTTGCTATCTTCTTC
NC021480_Ref_USA      TCTTACAATCCCTAGCGCTGGACACACCCCTCTTCTTACTGTTCAGTTGCTATCTTCTTC
GSV_BV4_PCR           ----------------------------------------------------TCTTCTTC
                                                                          ********

MF446638_TK           TTCTATTTCTCTTTTTAATTAGTGTAATATTTTGGTATTGTATTAGTATAAATAAAAAGA
KY211673_Iran         TTCTATTACTCTTTTTAATTGTTGTAATACTTTGGTATCGCATATATAGAAATAAAAAGA
NC021480_Ref_USA      TTCTAATTCTCTTTTTAATTATTATAATATTTTGGTATTGTCTTAATATAAATAAAAAGC
GSV_BV4_PCR           TTCTATTCCTCTTTATAACTATTATTATATTTTGGTATCATTTTAGTAAAAATATAAAGA
                      ***** * ****** *** *  * * *** ********    *   ** ***** ****

MF446638_TK           AAAAATCAGATGGTCAATCGC------CGTCCTAAAAATACTACGCGTCGTCGCGGGGTC
KY211673_Iran         AAAAATCGGATGGTTCCTCGACCTAATCGTCGGAACAATACATCGCGTCGTCGCGGGGTC
NC021480_Ref_USA      GGAAATCAGATGGTCAATCGC------CGTCCTAGAAATACTACGCGTCGTCGCGGGGTC
GSV_BV4_PCR           AAAAATCAGATGGTCAATAAC------CGTCGAAAAAATACTACGCGTCGTCGCGGGGTC
                        ***** ******   *         ****  *  *****  *****************

MF446638_TK           CCAGCTTTGCGGGAAGTGAATTACGCTACCGTAAACGCAGGGACGCAAACAGCATTCGCC
KY211673_Iran         CCAGCTTTGCGGGAAGTGAACTACGCTACCGTAAACGCAGGGACGCAAACAGCATTCGCC
NC021480_Ref_USA      CCAGCTTTGCGGGAAGTGAATTACGCTACCGTAAACGCAGGGACGCAAACAGCATTCGCC
GSV_BV4_PCR           CCAGCTTTGCGGGAAGTTAATTACGCTACCGTAAACGCAGGGACGCAAACAGCATTCGCC
                      ***************** ** ***************************************

MF446638_TK           AGGAAAGACCTTCAAATCTTAGCGGGCTGTGGAGACCGATCATTTAAATTGGTCGGCTTG
KY211673_Iran         AGGAAAGACCTTCAAATCTTAGCGGGCTGTGGAGATCGATCATTTAGGTTGGTCGGTTTA
NC021480_Ref_USA      AGGAAAGACCTTCAAATCTTAGCGGGCTGTGGAGACCGATCATTTAAATTGGTCGGCTTG
GSV_BV4_PCR           AGGAAAGACCTTCAAATCTTAGCGGGCTGTGGAGATCGATCATTTAAGTTGGTCGGCTTA
                      *********************************** **********  ******** ** 

MF446638_TK           GCTTTACAAGTCTCCTCTCTTTCAGAACCAGTTATTATACAGGTTCACATCTTCAACGAG
KY211673_Iran         GCTTTACAAATCTCCGCTCTTTCAGATCCAGTCATCGTGCAGGTTCACATCTTCAACGAG
NC021480_Ref_USA      GCTTTACAAGTCTCCTCTCTTTCAGAACCAGTTATCGTTCAGATTCACATCTTCAACGAG
GSV_BV4_PCR           GCTTTACAAGTCTCCTCTCTTTCAGAACCAGTCATCGTTCAGGTTCACATCTTCAACGAG
                      ********* ***** ********** ***** **  * *** *****************

MF446638_TK           TCTCTGAAAGAGATTGCGTTGTGCAACCGTTTGGTCGCCCAAGGTAACACTTGGTTGCGG
KY211673_Iran         GCACTGAAAGAGATTGCGTTGTGCAACCGTTTGGTCGCCCAAGGTAACACTTGGTTAAGG
NC021480_Ref_USA      GCACTGAAAGAGATTGCGTTGTGCAACCGTTTGGTCGCCCAAGGTAACACTTGGTTGCGG
GSV_BV4_PCR           GCACTGAAAGAGATTGCGTTGTGCAACCGTTTGGTCGCCCAAGGTAACACTTGGTTACGG
                       * *****************************************************  **

MF446638_TK           TTGCGCATCCCCCTTAGTTATAAGCAGTGGTGGTCCGGGGAGACCACTCAAAGTCAGACA
KY211673_Iran         TTGCGCATCCCCGTTAGTTATAAGCAGTGGTGGTCTGGGGAGACCACTCAAAGTCAGACA
NC021480_Ref_USA      TTGCGCATCCCCCTTAGTTATAAGCAGTGGTGGTCCGGGGAGACCACTCAAAGTCAGACA
GSV_BV4_PCR           TTGCGCATCCCCCTTAGTTATAAGCAGTGGTGGTCCGGGGAGACCACTCAAAGTCAGACA
                      ************ ********************** ************************

MF446638_TK           CTTGTCCGTGTCGATATTATTCCTACTTATAAAAATCAAAATACAAAAGTGTCTTTTTTA
KY211673_Iran         CTTGTCCGTGTCGATCTAATACCTACTTATAAAAATCAAAATTCAAAAGTGTCTTTTTTA
NC021480_Ref_USA      CTTGTCCGTGTCGATATTATACCTACTTATAAAAATCAAAATACAAAAGTGTCTTTTTTA
GSV_BV4_PCR           CTTGTCCGTGTCGATTTAATTCCTACTTATAAAAATCAAAATACAAAAGTGTCTTTTTTA
                      *************** * ** ********************* *****************

MF446638_TK           TTGAGTGTTTTGTGTCGGTTGGGCACAAGCGAGATAATAGCACACCCATGAAAGATTTGG
KY211673_Iran         TTGAGTGTTTTGTGTCGGTTGGGCACAAGCGAGATAACAGCACACCCATGAAAGATCTAG
NC021480_Ref_USA      TTGAGTCTTTTGTGTCGATTGGGCACAAGCGAGATAACAGCACACCCATGAAAGATTTAG
GSV_BV4_PCR           TTGAGTCTTTTGTGTCGGTTGGGCACAAGCGAGATAACAGCACACCCATGAAAGATCTAG
                      ****** ********** ******************* ****************** * *
```

```
MF446638_TK          TATTTTTCATGAGAAAAGGTAAACTTCGTCGATGACGATGTTTACGATTTTGTGATGGCC
KY211673_Iran        TATTTTTCATGAGAA-AGGTAAACTTCGTCGATGACGATGTTTACGATTTTGTGATGGCC
NC021480_Ref_USA     TATTTTTCATGAGAA-AGGTAAACTTCGTCGATGACGATGTTTACGATTTTGTGATGGCC
GSV_BV4_PCR          TATTTTTCATGAGAA-AGGTGAACTTCGTCGATGACGATGTTTACGATTTTGTGATGGCC
                     *************** **** ***************************************

MF446638_TK          CATTTTGTCAGTTGGGGGTTGGTACGGTGGAAACATTTGGTATCACTCTTCGATTTTGGC
KY211673_Iran        CACTATGTCAGTTGGGGGT-GGTACGGTGGAAACATC-GGCATCACCTTC-GACTTTGGT
NC021480_Ref_USA     CATTATGTCAGTTGGGGTT-GGTACGGTGGAAACATC-GGTATTACCTTC-GATTTTGGC
GSV_BV4_PCR          CACTATGTCAGTTGGGGGT-GGTACGGTGGAAACATC-GGCATCACCTTT-GATTTTGGC
                     ** * ************ * ***************** ** *****  *  ********

MF446638_TK          AATAAAATGTAAAGAATTTTTTAAATCGTGTTACTGATTCGTCAGCGGTAAGTAATAAAT
KY211673_Iran        AAT-AAGTGTAAAGAATTTTT-AAATCGTGTTACTGATCCGTCAGCGGTAAGTAATAAAT
NC021480_Ref_USA     AAT-AAATGTAAAGAATTTTT-AAATCGTGTTACTGATCCGTCAGCGGTAAGTAATAAAT
GSV_BV4_PCR          AAT-AAGTGTAAAGAATTTTT-AAATCGTGTTACTGATCCGTCAGCGGTAAGTAATAAAT
                     *** ** ************** **************************************

MF446638_TK          TTCTTCACAGTTGATAGCATTTAGCCGTCTCTGTGACACGAAATAGTATAAAGAATAAGA
KY211673_Iran        T--CTCACAGTTGATAGCATTTAGCCGTCTCTGTGACACG-AATAGTATTAAGAATAAGA
NC021480_Ref_USA     T--CTCACAGTTGATAGCATTTAGCCGTCTCTGTGACACG-AATAGTATAAAGAATAAGA
GSV_BV4_PCR          T--CTCACAGTTGATAGCATTTAGCCGTCTCTGTGACACG-AATAGTATTAAGAATAAGA
                     *   ************************************ ******** **********

MF446638_TK          TTATTGTAAGAAATTTTGATTTAATGTGAGTCCGGGTGGGCGCATGTCGTCTTAACTCAC
KY211673_Iran        TTATTGTAAGAAATTTTGACTTAATGTGAGTCCGGGTGGGCGCATGTCGTCTTAACTCAC
NC021480_Ref_USA     TTATTGTAAGAAATTTTGATTTAATGTGAGTCCGGGTGGGCGTATGTCGTCTTAACTCAC
GSV_BV4_PCR          TAATTGTAAGAAATTTTGACTTAATGTGAGTCCGGGTGGGCGTATGTCGTCTTAACTCAC
                     * ***************** ********************** *****************

MF446638_TK          CCGTAAAGGTA-TCTTAACATAGTTATGAATAAGTCCTAGTTAG
KY211673_Iran        CCGTAAAGGTTG-CTTAACTTAGTTATGTGTAAGTCCTAGTTAG
NC021480_Ref_USA     CCGTAAAGGT-GTCTTAACATAGTTATGAATAAGTCCTAGTTAG
GSV_BV4_PCR          CCGTTAAGGTTGT-TTAACATAG-TATGA----------------
                     **** *****   ********** ****
```

Supplementary Figure 6

Multiple sequence alignment of GSV of the sequences in the GenBank and isolate of 1_TK and 13_BV (Individual BV4) prepared by Clustal omega program. Grey boxes show SNPs present only in the Reference, whereas green boxes highlight SNPs in the other strains.

```
NC_001920_Ref_USA     ACCCCTGCAAAGAGGTCCTCGGATCACTTTCC-TGTGGTTCCTGTGGTTACACCTCGGAAGGCCGCCG
KU668664_Brasil       ACCCCCGCTAAGAGGTCTCCGGATCTTCTTGCTTGTGGTTCCTGTGGTTTCACCTCGGAAGGCCGCCG
AB742222_India        ACCCCCGCTAAGAGGTCTCCGGATCTTCTTGCTCGTGGTTCCTGTGGTTTCACCTCGGAAGGCCGCCG
JF746184_China        ACCCCCGCTAAGAGGTCTCCGGATCTTCTTGCTTGTGGTTCCTGTGGTTACACCTCGGAAGGCCGCCG
KT000348_Czech        ACCCCCGCTAAGAGGTCTCCGGATCTTCTTGCTTGTGGTTCCTGTGGTTTCACCTCGGAAGGCCGCCG
X06904_Australia      ACCCCCGCTAAGAGGTCTCCGGATCTTCTTGCTTGTGGTTCCTGTGGTTTCACCTCGGAAGGCCGCCG
X87911_Germany        ACCCCCGCTAAGAGGTCTCCGGATCTTCTTGCTTGTGGTTCCTGTGGTTTCACCTCGGAAGGCCGCCG
JN008866_Iran         ACCCCCGCTAAGAGGTCTCCGGATCTTCTTGCTTGTGGTTCCTGTGGTTTCACCTCGGAAGGCCGCCG
MF510389_HT           ACCCCCGCTAAGAGGTCTCCGGATCTTCTTGCCTGTGGTTCCTGTGGTTTCACCTCGGAAGGCCGCCG
MF510390_PP           ACCCCCGCTAAGAGGTCTCCGGATCTTCTTGCCTGTGGTTCCTGTGGTTTCACCTCGGAAGGCCGCCG
MF510391_ET           ACCCCCGCTAAGAGGTCTCCGGATCTTCTTGCCTGTGGTTCCTGTGGTTTCACCTCGGAAGGCCGCCG
                      ***** ** ********  ******   ** *  **********************************

GYSVd1.PN:16/21                                                     A..A
NC_001920_Ref_USA     CGGACCTGCAAAGAA-GAAGATAGGGGCAGAGGGGGAGTGAGCCTCGTCGTCGACGAAGGG
KU668664_Brasil       CGGACCTGCAAAGAA-GAAGATAGGGGCAGAGGGGGAGTGAGCCTCGTCGTCGACGAAGGG
AB742222_India        CGGACCTGCAAAGAA-GAAGATAGGGGCAGAGGGGGAGTGAGCCTCGTCGTCGACGAAGGG
JF746184_China        CGGACCTGCAGAGAA-GAAGATAGGGGCAGAGGGGGAGTGAGCCTCGTCGTCGACGAAGGG
KT000348_Czech        CGGACCTGCAAAGAA-GAAGATAGGGGCAGAGGGGGAGTGAGCCTCGTCGTCGACGAAGGG
X06904_Australia      CGGACCTGCAAAGAA-GAAGATAGGGGCAGAGGGGGAGTGAGCCTCGTCGTCGACGAAGGG
X87911_Germany        CGGACCTGCAAAGAA-GAAGATAGGGGCAGAGGGGGAGTGAGCCTCGTCGTCGACGAAGGG
JN008866_Iran         CGGACCTGCAAAGAA-GAAGATAGGGGCAGAGGGGGAGTGAGCCTCGTCGTCGACGAAGGG
MF510389_HT           CGGACCTGCAAAGAA-GAAGATAGGGGCAGAGGGGGAATGAGCCTCGTCGTCGACGAAGGG
MF510390_PP           CGGACCTGCAGAGAA-GAAGATAGGGGCAGAGGGGGTTTGAGCCTCGTCGTCGACGAAGGG
MF510391_ET           CGGACCTGCAAGAAAAGAAGATAGGGGCAGAGGGGGT-TGAGCCTCGTCGTCGACGAAGGG
                      **********   ** ********************  ***********************

NC_001920_Ref_USA     GTGCACTCCAAAGCTCCGAACTGGCGTC-GTCCGGCTCTCCTCGGAGCCTCGCTGCTCTGG
KU668664_Brasil       GTGCACTCCGAGTGCCTGAGCTGGTCGACGTCCAGCTCCCTCGGGAC-CTGC-TGCTCTGG
AB742222_India        GTGCACTCCGAGTGCCTGAGCTGGTCGACGTCCAGCTCCCTCGGGAC-CTGT-TGCTCTGG
JF746184_China        GTGCACTCCGAGTGCCTGAGCTGGTCGACGTCCAGCTCCCCCGGGACCACGC-TGCTCTGG
KT000348_Czech        GTGCACTCCGAGTGCCTGAGCTGGTCGACGTCCAGCTCCCTCGGGACCACGC-TGCTCTGG
X06904_Australia      GTGCACTCCGAGTGCCTGAGCTGGTCGACGTCCAGCTCCCTCGGGACCACGC-TGCTCTGG
X87911_Germany        GTGCACTCCGAGTGCCTGAGCTGGTCGACGTCCAGCTCCCTCGGGACCACGC-TGCTCTGG
JN008866_Iran         GTGCACTCCGAGTGCCTGAGCTGGTCGACGTCCAGCTCCCTCGGGACCACGC-TGCTCTGG
MF510389_HT           GTGCATTCCGAGTGCCTGAGCTGGTCGACGTCCAGCTCCCTCGGGACCACGC-TGCTCTGG
MF510390_PP           GTGCAAACCGAGTGCCTGAGCTGATCGACGTCCAGCTCCCTCGGGACCACGC-TGCTCTGG
MF510391_ET           GTGCAATCCGAGTGCCTGAGCTGGTCGACGTCCAGCTCCCTCGGGACCACGC-TGCTCTGG
                      *****  ** *    * ** ***      **** ****  *  **** *    ********

NC_001920_Ref_USA     GCGGAAGAGTCTTCTGACTTTTCTAGCCTATTCAGCATTGCGCTCTTGAGGCCCGGCGAA
KU668664_Brasil       GCGGAAGAGTCTTCTGACACTGCTAGCCTATTCAGCTTCGCGTCCTTGAGGCCTGGCGTA
AB742222_India        GCGGAAGAGTCTTCTGACACTTCTAGCCTATTCAGCTTCGCGTCCTTGAGGCCTGGCGTA
JF746184_China        GCGGAAGAGTCTTCTGACACTTCTAGCCTATTCAGCTTCGCGTCCTTGAGGCCTGGCGTA
KT000348_Czech        GCGGAAGAGTCTTCTGACACTTCTAGCCTATTCAGCTTCGCGTCCTTGAGGCCTGGCGTA
X06904_Australia      GCGGAAGAGTCTTCTGACACTTCTAGCCTATTCACGTTCGCGTCCTTGAGGCCTGGCGTA
X87911_Germany        GCGGAAGAGTCTTCTGACACTTCTAGCCTATTCAGCTTCGCGTCCTTGAGGCCTGGCGTA
JN008866_Iran         GCGGAAGAGTCTTCTGACTTTTCTAGCCTATTCAGCTTCGCGTCCTTGAGGCCTGGCGTA
MF510389_HT           GCGGAAGAGTCTTCTGACTTTTCTAGCCTATTCAGCTTCGCGTCCTTGAGGCCTGGCGTA
MF510390_PP           GCGGAAGAGTCTTCTGACACTTCTAGCCTAATCAGCTTCGCGTCCTTGAGGCCTGGCGTA
MF510391_ET           GCGGAAGAGTCTTCTGACACTTCTAGCCTATTCAGCTTCGCGTCCTTGAGGCCTGGCGTA
                      ******************  * ******** ***   * ***  ********* **** *

NC_001920_Ref_USA     ACGCGGTTCGTCTGCTGAGGATGCCTCCGCTAGTCGAGCGGACTTGGTCTCTTCC-GCCC
KU668664_Brasil       ACGCGGCTCTTGCCACCAGGATGCCTCCGCTAGTCGAGCGGACTTGGTCTCTTCC-GCCC
AB742222_India        ACGCGGCTCTTGCCACCAGGATGCCTCCGCTAGTCGAGCGGACTTGGTCTCTTCC-GCCC
JF746184_China        ACGCGGCTCTTGCCACCAGGATGCCTCCGCTAGTCGAGCGAACTTGGTCTCTTCC-GCCC
KT000348_Czech        ACGCGGCTCTTGCCACCAGGATGCCTCCGCTAGTCGAGCGGACTTGGTCTCTTCC-GCCC
X06904_Australia      ACGCGGCTCTTGCCACCAGGATGCCTCCGCTAGTCGAGCGGACTTGGTCTCTTCC-GCCC
X87911_Germany        ACGCGGCTCTTGCCACCAGGATGCCTCCGCTAGTCGAGCGGACTTGGTCTCTTTCCGCCC
JN008866_Iran         ACGCGGCTCTTGCCACCAGGATGCCTCCGCTAGTCGAGCAGACTTGGTCTCTTTCCGCCC
MF510389_HT           ACGCGGCTCTTGCCACCAGGAAGCCTCCGCTAGTCGAGCGGACTTGGTCTCTATCCGCCC
MF510390_PP           ACGCGGCTCTTGCCACCTGGCTGCCTCCGCTAGTCGAGCGGACTTGGTCTCTTCC-GCCC
MF510391_ET           ACGCGGCTCTTGCCACCAGGAAGCCTCCGCTAGTCGAGCGGACTTGGTCTCTTCC-GCCC
                      ****** ** *       **  *****************  ***********  * ****
```

```
NC_001920_Ref_USA      AAAGCCCTTTTTCTTTCAACTGAGCTTGTTCCAACGCGCCCCGCGAGTGCAATCCCCGGA
KU668664_Brasil        AAAGCCCTTTTTCTTTCAACTGAGCTTGTTCCAACGCGCCCCGCGAGTGCAATCCCCGGA
AB742222_India         AAAGCCCTTTTTCTTTCAACTGAGCTTGTTCCAACGCGCCCCCCGAGTGCAATCCCCGGA
JF746184_China         AAAGCCCTTTTTCTTTCTAATCAGCTTGTTCCAACGCGCCCCGCGAGTGCAATCCCCGGA
KT000348_Czech         AAAGCCCTTTTTCTTTCAACTGAGCTTGTACCAACGCGCCCCGCGAGTGCAATCCCCGGA
X06904_Australia       AAAGCCCTTTTTCTTTCAACTGAGCTTGTTCCAACGCGCCCCGCGAGTGCAATCCCCGGA
X87911_Germany         AAAGCCCTTTTTCTTTCAACTGAGCTTGTACCAACGCGCCCCGCGAGTGCAATCCCCGGA
JN008866_Iran          AAAGCCCTTTTTCTTTCAACTGAGCTTGTTCCAACGCGCCCCGCGGGTGCAATCCCCGGA
MF510389_HT            AAAGCCCTTTTTCTTTCAACTGAGCTTGTACCAACGCGCCCCGCGAGTGCAATCCCCGGA
MF510390_PP            AAAGCCCTTTTTCTTTCTAATCAGCTTGTTCCAACGCGCCCCGCGAGTGCAATCCCCGGA
MF510391_ET            AAAGCCCTTTTTCTTTCAATTCTGCTTGTTCCAACGCGCCCCGCGAGTGCAATCCCCGGA
                       ***************** * *  ****** ************ ** **************
```

Supplementary Figure 7

Multiple sequence alignment of different GYSVd1 variants including Hungarian isolates prepared by Clustal omega program. Grey boxes show SNPs present only in the Reference whereas green boxes highlight SNPs in the other strains.
